# Computational modelling for improved translation of cardiac inotropic and lusitropic drug effects from rats to humans^☆^

**DOI:** 10.1016/j.vascn.2025.107747

**Published:** 2025-05-13

**Authors:** Alexander Jung, Christoph M. Augustin, Julia Voglhuber-Höller, Mara Kiessling, Senka Ljubojevic-Holzer, Gary R. Mirams, Steven A. Niederer, Gernot Plank

**Affiliations:** a Gottfried Schatz Research Center for Cell Signaling, Metabolism and Aging – Division of Medical Physics and Biophysics, Medical University of Graz, Graz, Austria; b BioTechMed-Graz, Graz, Austria; c Department of Cardiology, Medical University of Graz, Graz, Austria; d Gottfried Schatz Research Center for Cell Signaling, Metabolism and Aging – Division of Molecular Biology and Biochemistry, Medical University of Graz, Graz, Austria; e Centre for Mathematical Medicine & Biology, School of Mathematical Sciences, University of Nottingham, Nottingham, United Kingdom; f Division of Imaging Sciences & Biomedical Engineering, King’s College London, London, United Kingdom

**Keywords:** Cardiovascular pharmacology, Computer simulation, In silico modelling, Drug discovery, Cardiac safety, 3Rs

## Abstract

Telemetered rats are widely used for early drug screenings but pronounced physiological differences between rat and human hearts limit translational relevance. To address this, the study investigates the potential of computer modelling to improve the translation of inotropic and lusitropic drug effects from rats to humans, beginning at the cellular scale. To this end, computer models of rat and human left ventricular cardiomyocytes were constructed to reproduce experimental data. First, global sensitivity analyses identified distinctive differences in inotropic and lusitropic responses to the inhibition of ion channels and transporters in rats and humans. Then, the computer models were used to address the translation challenge by predicting human responses based on sarcomere length and intracellular [Ca^2+^] data obtained from rats. This process, referred to as computational drug effect translation, involved identifying the drug’s blocking potencies on potential targets. Focussing on the identifiable targets RyR2, SERCA2, and NCX1, evaluations on synthetic data showed high translation accuracy across all biomarkers and drug concentrations. For example, coefficients of determination were ≥ 0.997 for predicted human effects compared to ≤0.771 for rat effects for percentage sarcomere shortening, and ≥ 0.905 compared to ≤0.418 for the time from peak to 90 % relaxation. Evaluations on experimental data collected for thapsigargin largely corroborated these findings. The results demonstrate that computer modelling can improve the translation of inotropic and lusitropic drug effects from rats to humans, offering potential benefits for augmenting the current drug development pipeline.

## 1. Introduction

Myocardial contractility (inotropy) and relaxation (lusitropy) are pivotal for heart function while impairments can have severe cardiovascular consequences (Brutsaert, Sys, & Gillebert, 1993; Guth et al., 2019). Therefore, increasing efforts are directed towards the discovery and development of efficacious inotropic and lusitropic drugs (Hwang & Sykes, 2015), and the minimisation of unintended drug effects that adversely impact inotropy and lusitropy (Guth et al., 2019; Pagell et al., 2016). The conscious telemetered rat model is popular for drug efficacy and safety screenings because it enables high throughput with low resource intensity, and left ventricular pressure in rat hearts is similar to that in human hearts (Accardi et al., 2016; Guth, 2007; Milani-Nejad & Janssen, 2014). However, pronounced physiological differences between rats and humans exist across multiple scales, manifesting for instance in faster heart rates, and faster contraction and relaxation dynamics in rat hearts (Milani-Nejad & Janssen, 2014; Stevenson-Cocks, 2019). These differences can result in distinct quantitative and even qualitative drug responses (e.g. in itraconazole: Ahmad, Singer, & Leissa, 2001; Accardi et al., 2016), hampering the translation of rat derived inotropic and lusitropic drug effects to human. Inaccurate translations are a major problem in drug development, leading to premature elimination of potentially useful drug candidates or high attrition rates in clinical trials due to adverse events that may harm participants (Cook et al., 2014; Van Norman, 2019).

Cardiac computer modelling has emerged as promising tool to understand and address existing translation gaps. Cardiomyocyte computer models, in particular, have demonstrated maturity in mechanistic detail and the reproduction of experimental findings, leading to an increasing number of pharmacological applications in academia and beyond (Aghasafari et al., 2021; Beattie et al., 2013; Fogli Iseppe et al., 2021; Forouzandehmehr, Paci, Koivumäki, & Hyttinen, 2022; Gong & Sobie, 2018; Grandits et al., 2024; Jæger et al., 2020; Li et al., 2019; Margara et al., 2021; Mirams et al., 2011; Mirams et al., 2014; Moreno, Zhu, Mangold, Chung, & Silva, 2019; Morotti et al., 2021; Niederer, Lumens, & Trayanova, 2019; Passini et al., 2017; Passini et al., 2021; Trovato et al., 2025; Tveito et al., 2018; Tveito, Jæger, Maleckar, Giles, & Wall, 2020; Yang et al., 2020; Yang & Clancy, 2012). Several works have recently demonstrated the potential of cardiomyocyte computer models to improve the translation of drug effects on the action potential and intracellular [Ca^2+^] transients from various animal cardiomyocytes and human induced pluripotent stem cell (hiPSC)-derived cardiomyocytes to human ventricular cardiomyocytes (Aghasafari et al., 2021; Gong & Sobie, 2018; Jæger et al., 2020; Morotti et al., 2021; Tveito et al., 2018; Tveito et al., 2020). However, to the best of our knowledge, no work has been published that has explored the potential of cardiomyocyte computer models for improving the translation of inotropic and lusitropic drug effects.

Focusing first on the cellular scale, we hypothesise that computer modelling can improve the translation of inotropic and lusitropic drug effects from rats to humans. To test this hypothesis, we constructed computer models of rat and human ventricular cardiomyocytes and utilised experimental data for their recalibration and evaluation. First, we conducted global sensitivity analyses using these models to identify different inotropic and lusitropic responses to the inhibition of target proteins present in both humans and rats. Then, we developed an integrated computational approach for predicting drug effects in human ventricular cardiomyocytes based on measurements of sarcomere length and intracellular [Ca^2+^] in rat ventricular cardiomyocytes. This approach was evaluated using synthetic and experimental data.

## 2. Materials and methods

### 2.1. Transients and biomarkers

**Fig. 1.**
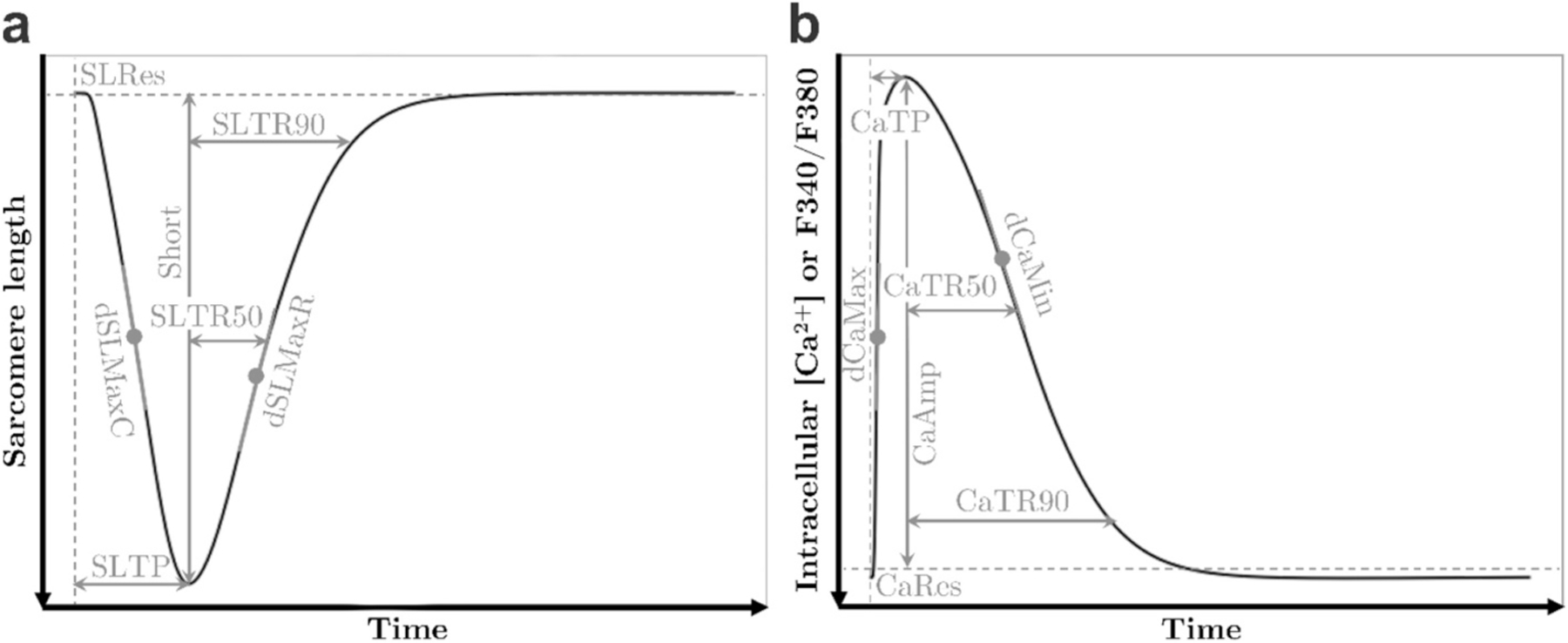
Transients and definition of characteristic biomarkers. (**a**) sarcomere length, (**b**) experiments: fluorescence intensity ratio (F340/F380), simulations: intracellular [Ca^2+^]. Sarcomere length biomarkers include the resting value (SLRes), the time from stimulus to peak (SLTP), percentage sarcomere shortening (Short) defined as amplitude relative to the resting value expressed as percentage, the times from peak to 50 % (SLTR50) and 90 % return of the transient (SLTR90), the maximum rate during contraction from stimulus to peak (dSLMaxC), and the maximum rate during relaxation from peak onwards (dSLMaxR). Intracellular [Ca^2+^] biomarkers include the resting value (CaRes), the time from stimulus to peak (CaTP), the amplitude (CaAmp), the time from peak to 50 % (CaTR50) and 90 % return of the transient (CaTR90), the maximum rate from stimulus to peak (dCaMax), and the minimum rate from peak onwards (dCaMin).

Inotropy and lusitropy were investigated based on sarcomere length transients in unloaded left ventricular cardiomyocytes. To address the diverse mechanisms underlying inotropic and lusitropic drug effects within the coupled intracellular Ca^2+^ handling and contraction-relaxation processes, intracellular [Ca^2+^] transients were also considered. Throughout the study, we utilised seven biomarkers for each transient to characterise the key features of the phases between stimulus and peak, and between peak and return (Fig. 1). The percentage sarcomere shortening (Short) and relaxation times (SLTR50 and SLTR90) were particularly examined for assessing inotropy and lusitropy, respectively. While the biomarkers for experimental transients were extracted from an averaged beat (**2.3.2**), the biomarkers of simulated transients were extracted from the last beat. Corresponding drug effects were defined as percentage changes between the values before (baseline) and after drug administration.

Transients exhibiting alternans or fluctuations were deemed abnormal. In experimental transients, alternans and fluctuations were visually detected, while in simulated transients, they were identified within the last two beats using specific thresholds. Alternans was identified using a beat-to-beat amplitude ratio > 1.1 (Morotti et al., 2021), and fluctuations were defined as rates >0.001 μm/ms before the peak and < −0.001 μm/ms after the peak in sarcomere length transients, and as rates of < −0.001 μM/ms before the peak and > 0.001 μM/ms after the peak in intracellular [Ca^2+^] transients.

### 2.2. Proteins and drug action

Ten proteins including ion channels, pumps, and exchangers (Table 1) were of interest in this study for their role as potential drug targets. These proteins were selected from ion channels and transporters for which drug effects are well characterised and that are represented in either the rat or the human left ventricular cardiomyocyte computer model. This study exclusively considered inhibitory drug effects.

### 2.3. Experiments

Transients of sarcomere length and intracellular [Ca^2+^] were collected in unloaded isolated rat left ventricular cardiomyocytes. The experiments were conducted at the Medical University of Graz (Graz, Austria) and all procedures were carried out in accordance with the Federal Act on the Protection of Animals and according to European ethical regulations.

#### 2.3.1. Animals and cardiomyocyte isolation

Healthy Sprague Dawley and Wistar rats between 18 and 81 weeks (Table S1) were acquired either through the 3R in action program of the Medical University of Graz or purchased from Janvier Labs (France). The rats were housed in a conventional facility in small groups of up to three per cage (littermates of the same sex), in adequately sized open cages. Absorbent wood-based bedding and nesting material were provided and frequently changed. Wood pieces were offered as enrichment, and rat houses were placed within the cages. Fresh water and food were provided daily. Trained animal carers carried out daily tasks, and veterinarians were available on site in case of any concern or illness. None of the rats presented with health issues, hence, no humane endpoints are described in detail. For organ collection, the rats were sacrificed by a high dose of isoflurane inhalation anaesthesia (96 % O_2_, 4 % Isoflurane) followed by cervical dislocation.

For cardiomyocyte isolation, a liberase-based Langendorff perfusion protocol was employed (Matzer et al., 2022). In brief, after opening the thoracic cavity, the hearts were rapidly excised and submerged in ice-cold perfusion buffer (PB, in mM: 135 NaCl, 4.7 KCl, 0.6 KH_2_PO_4_, 0.6 HNa_2_PO_4_ dibasic, 1.2 MgSO_4_*7H_2_O, 10 HEPES, 30 Taurin, 2,3-butanedione monoxime, 10 Glucose, pH 7.4) with 1 mM CaCl_2_ added. The aorta was cannulated for retrograde perfusion. After 1.5 min of perfusion with a pre-warmed (37 °C) Ca^2+^-free solution (PB), the hearts were digested in a liberase-based approach (PB added: 0.037 mM CaCl2, 0.014 % trypsin, 0.15 mg ml^−1^ liberase; Liberase^™^ Research Grade, Merck, Germany) for 9–12 min. Digestion was stopped by submerging the ventricles in a bovine calf serum (BCS)-containing stop solution (PB added in mM: 0.014 CaCl_2_, 11 % BCS) and the tissue was carefully dissociated using a cut and blunted Pasteur pipette followed by sieving through a 300 μm mesh. The cardiomyocytes were allowed to settle by gravity before being exposed to a series of Ca^2+^ dilutions (PB added: 5.2 % BCS, CaCl_2_ in 5 steps) reaching a maximum of 1.5 mM CaCl_2_.

#### 2.3.2. Measurement of sarcomere length and intracellular [Ca^2+^]

Sarcomere length was measured video-based using the *SarcLen Sarcomere Length Acquisition module* (MyoCam-S, IonOptix, The Netherlands) and images were recorded at an acquisition rate of 500 Hz. Intracellular [Ca^2+^] was measured as fluorescence intensity ratio using the ratiometric dye fura-2 (fura-2, AM, cell-permeant; invitrogen^™^ F1221; Thermo Fisher Scientific, USA). To this end, fura-2 was diluted in DMSO according to the manufacturer’s instructions and stored in aliquots at −20 °C until further usage. The cardiomyocytes were then stained with a final concentration of 2 μM in a total volume of 1 ml NT in the dark for a total of 20 min with several inverting steps. After two washing steps with NT and a de-esterification period of a minimum of 15 min, fluorescence measurements were acquired using a Nikon Eclipse Ti2 epifluorescence microscope equipped with a Nikon Super Fluor 40×/0.9 N.A. objective (Ljubojevic-Holzer et al., 2020, 2022). Fura-2 was excited at 340 and 380 nm using a HyperSwitch Dual Excitation Light Source (IonOptix, The Netherlands) and emission at 512 nm was recorded at an acquisition rate of 1 kHz. To avoid negative inotropic effects of fura-2 (Robinson et al., 2023), sarcomere length measurements were conducted separately. Although different cardiomyocytes were used for the sarcomere length and intracellular [Ca^2+^] measurements, they were isolated from the same hearts to ensure comparability.

Measurements were conducted at 35–37 °C and cardiomyocytes were paced by field stimulation using monophasic pulses of 12.05 mV/cm for 5 ms. The maximum pacing frequency used in the experiments was 2 Hz, which is much lower than typical heart rates in rats (Milani-Nejad & Janssen, 2014). This lower frequency was chosen to ensure sufficient data collection, as not all cardiomyocytes were responsive to higher pacing frequencies due to their reduced stress tolerance when isolated from the physiological syncytium network. For each experimental condition, pacing was applied to the cardiomyocytes until the transients showed no visible differences indicating that a limit cycle had been approached. Then, the transients from the subsequent 20 s were averaged and biomarkers were determined based on this averaged transient (**2.1**). Transient acquisition and determination of biomarker values were performed using *IonWizard* (version 7.5.3; IonOptix, The Netherlands).

#### 2.3.3. Administration of thapsigargin

Thapsigargin (T9033–5MG, Sigma Aldrich, USA) was dissolved in DMSO (stock concentration 10 mM, stored in aliquots at −20 °C) and further diluted in NT to yield the four concentrations 0.1, 1, 10 and 30 μM. To avoid any vehicle-related effects, the final DMSO concentration was kept constant at 40 mM in all solutions used. The concentrations of thapsigargin were selected in line with Abi-Gerges et al. (2020) who published drug effects on sarcomere length biomarkers in human ventricular cardiomyocytes. These data were used for the evaluation of the computational drug effect translation (**2.6.7**).

#### 2.3.4. Data sets and exclusion criteria

Three independent sarcomere length and fluorescence intensity ratio data sets were generated (Table S1). Data set #1 was collected at a pacing frequency of 1 Hz. Data set #2 was collected at the pacing frequencies 0.5, 1, and 2 Hz. Data set #3 was collected at a pacing frequency of 1 Hz before and after thapsigargin administration at four concentrations. For some cardiomyocytes, data could not be collected under all experimental conditions due to recording issues or cell death and data were excluded if abnormalities were detected in the corresponding transients (**2.1**). In data set #2, all data produced by a given cardiomyocyte were excluded if, at any pacing frequency, data either could not be collected or were excluded.

#### 2.3.5. Comparison of data and simulations

Experimental sarcomere length data were compared to simulated sarcomere lengths. However, since fluorescence intensity ratios were not transformed into calibrated intracellular [Ca^2+^], a direct comparison to simulated intracellular [Ca^2+^] was not possible. Consequently, model recalibration based on data set #1 was performed using only the sarcomere length data. Nevertheless, measured and simulated drug effects on both sarcomere length and intracellular [Ca^2+^] biomarkers were compared, with only a slight inaccuracy for the latter.

### 2.4. Computer models

#### 2.4.1. Model description

Computer models of rat and human left ventricular cardiomyocytes were constructed based on established model components sourced from the existing literature that were developed using data collected in left ventricular cardiomyocytes at 37 °C. The models account for electrophysiological, Ca^2+^ handling, and contraction-relaxation processes. Electrophysiological and Ca^2+^ handling processes in rat and human cardiomyocytes are represented by the (data-driven 1 Hz variant of the) Gattoni model (Gattoni et al., 2016) and the ToR-ORd-dynCl model (Tomek et al., 2019; Tomek, Bueno-Orovio, & Rodriguez, 2020), respectively. Contraction-relaxation processes in rat and human cardiomyocytes are represented by the same model component, the Land model (Land et al., 2017), following the assumption that the involved proteins differ in density, structure and function, but are present in both rat and human cardiomyocytes associated with the conservation of fundamental physiological mechanisms (Tøndel, Land, Niederer, & Smith, 2015). The Land model has two components that describe the active and passive stress generation, respectively, and based on the stress equilibrium, sarcomere length transients were computed in line with Jung, Gsell, Augustin, and Plank (2022). Initial sarcomere lengths were set to the median resting sarcomere lengths obtained experimentally (Table 2). This assumes full relaxation of the sarcomeres during data collection, which was, however, not tested. The bidirectional interaction of electrophysiological, Ca^2+^ handling, and contraction-relaxation processes through excitation-contraction coupling and mechano-electrical feedback were accounted for in line with previous work (Levrero-Florencio et al., 2020; Timmermann et al., 2017). In detail, the free intracellular [Ca^2+^] obtained from the Ca^2+^ handling model component (rat: Gattoni model, human: ToR-ORd-dynCl) is input for the contraction-relaxation model component (Land model) to determine the fraction of troponin C units with intracellular Ca^2+^ bound to its regulatory binding site. Then, the intracellular [Ca^2+^] bound to troponin C is computed and fed back to the Ca^2+^ handling model to update the free intracellular [Ca^2+^]. To accommodate this, the free intracellular [Ca^2+^] in the rat cardiomyocyte model is computed as

$$\begin{array}{l} \frac{d{\left[Ca^{2+}\right]}_{i}}{dt}=\beta_{Cai}\left[-\left(-2I_{NCX}+I_{pCa}+I_{Cab}+I_{LCC}\right)\frac{1}{2Fv_{myo}}\right.+J_{RYR}-J_{SERCA}\\ \;\;\;\;\;\;\;\;\;\;\;\;\;\;\;\;\;\;\;\;\;\;\;\;\;\;\;\left.+J_{SRl}-\frac{d{\left[Ca^{2+}\right]}_{TRPN}}{dt}\right], \end{array}$$

and the free intracellular [Ca^2+^] in the human cardiomyocyte model is computed as

$$\begin{array}{l} \frac{d{\left[Ca^{2+}\right]}_{i}}{dt}=\beta_{Cai}\left[-\left(-2I_{NaCa}+I_{pCa}+I_{Cab}+I_{CaL,i}\right)\frac{A_{cap}}{2Fv_{myo}}\right.-J_{up}\frac{v_{nsr}}{v_{myo}}\\ \;\;\;\;\;\;\;\;\;\;\;\;\;\;\;\;\;\;\;\;\;\;\;\;\;\;\;\left.+J_{\text{d}iff,Ca}\frac{v_{ss}}{v_{myo}}-\frac{d{\left[Ca^{2+}\right]}_{TRPN}}{dt}\right], \end{array}$$

where

$$\beta_{Cai}=\frac{1}{1+\frac{\left[\bar{CMDN}\right]K_{CMDN}}{{\left({\left[Ca^{2+}\right]}_{i}+K_{CMDN}\right)}^{2}}}.$$

relates to the calmodulin buffering. The intracellular [Ca^2+^] bound to troponin C is computed as

$$\frac{d{\left[Ca^{2+}\right]}_{TRPN}}{dt}=\bar{{\left[Ca^{2+}\right]}_{TRPN}}\frac{dCaTRPN}{dt}$$

where

$$\frac{dCaTRPN}{dt}=k_{TRPN}\left[{\left(\frac{{\left[Ca^{2+}\right]}_{i}}{{\left[Ca^{2+}\right]}_{T50}^{ref}}\right)}^{n_{TRPN}}\left(1-CaTRPN\right)-CaTRPN\right]$$

is the fraction of troponin C units with intracellular Ca^2+^ bound to its regulatory binding site and $\bar{{\left[Ca^{2+}\right]}_{TRPN}}$ is the maximum [Ca^2+^] that can bind to troponin C.

The subcellular drug effect on a given protein of interest p (Table 1) was modelled using the pharmacological scaling factor $s_{p}$ that was applied to the corresponding baseline ion current $I_{p}^{base}$:

$$I_{p}^{drug}=I_{p}^{base}s_{p}.$$

Since drug action was limited to inhibition, the range of pharmacological scaling factors was from 0 to 1, with smaller values indicating stronger inhibitory effects. The Hill equation establishes a relationship between the pharmacological scaling factor and the drug concentration C (in μM) accounting for the drug’s blocking potency for the given protein and cooperativity of the drug binding:

$$s_{p}=\frac{1}{1+{\left(\frac{C}{IC50_{p}}\right)}^{h_{p}}}.$$

The half-maximal inhibitory concentration $IC50_{p}$ represents the drug’s blocking potency for the given protein, and $h_{p}$ is the Hill coefficient representing the cooperativity of the drug binding. The ranges for these parameters are 0 to ∞ μM and 0 to ∞, respectively, and while larger $IC50_{p}$ values indicate smaller blocking potency, $h_{p}<1$ indicates negative cooperativity, $h_{p}=1$ indicates non-cooperative binding, and $h_{p}>1$ indicates positive cooperativity.

#### 2.4.2. Forward simulations

The computer models were implemented in both the Cardiac Arrhythmia Research Package *CARPentry* (Vigmond, Weber dos Santos, Prassl, Deo, & Plank, 2008) and the freely available *openCARP* (Plank et al., 2021). Implementations were based on the published *CellML* (Gattoni model: Gattoni_1Hz.cellml, https://models.cellml.org/workspace/285; ToR-ORd-dynCl model: ToRORd_dynCl_endo.cellml, https://github.com/jtmff/torord/tree/master/cellml) or *Matlab* files (Land model: https://www.cemrg.co.uk/models) with the modifications described above. Forward simulations were performed with the single-cell tool *bench* using a combination of the Rush-Larsen method (Rush & Larsen, 1978) and the forward Euler method. Specifically, the Rush-Larsen method, was utilised to solve for the gating variables of the electrophysiological model component, whereas the forward Euler method was utilised to solve for the other variables. The solver time steps were 0.01 ms and the simulated transients were saved at a sampling interval of 1 ms.

Extracellular ion concentrations before model recalibration were those provided in the published model files, while after recalibration, they were set in alignment with the sarcomere length measurements (Table S2). When comparing simulations with experimental data, extracellular ion concentrations were set to match the respective experiments unless otherwise noted. Stimuli were applied as monophasic pulses with 50 μA/cm^2^ for 1 ms at a frequency of 1 Hz. The stimulus time integral 50 μAms/cm^2^ was always maintained for monophasic stimuli by adjusting the stimulus strength for the given stimulus duration, and this was similarly applied to counter stimuli in biphasic stimuli (−50 μAms/cm^2^).

Starting from the initial states provided in the published model files, a series of 100 stimuli were applied to the computer models to approach the limit cycle for each new parameter set introduced. This small number of stimuli was chosen to keep the computational cost tractable but the peaks of intracellular [Na^+^] and [Ca^2+^], and sarcomere length changed only by 1.2 %, 0.8 % and − 0.2 % (rat cardiomyocyte model), and − 3.1 %, −1.3 % and 0.2 % (human cardiomyocyte model), respectively, when the number of stimuli was increased to 1000, using the recalibrated models (at baseline).

#### 2.4.3. Model recalibration

The computer models were recalibrated using available experimental sarcomere length and intracellular [Ca^2+^] biomarker data (Table S2). This was done to account for the differences between rat and human excitation-contraction coupling and mechano-electrical feedback. Initially, all parameters of the contraction-relaxation model component, along with one major parameter (maximum conductances or similar) related to each protein of interest represented in both the rat and the human cardiomyocyte model (Table 1), and the maximum [Ca^2+^] that can bind to troponin C were considered for recalibration (Table S3). Global sensitivity analyses (Section 2.5) were performed to determine parameters to which all experimental biomarkers are insensitive. These parameters were deemed non-identifiable (Guillaume et al., 2019) and thus excluded from the recalibration. Through this process, the number of parameters considered for recalibration was reduced from 27 to 9 in the rat cardiomyocyte model and from 27 to 11 in the human cardiomyocyte model (Fig. S1). Notably, the lists of considered parameters in both models includes the same parameters of the contraction-relaxation model component (Table 3).

The recalibrated values for these parameters were found by minimising the following objective function:

$$Cost=\frac{1}{11}\left[\sum_{k=1}^{7}{\left(\frac{B_{SL,k}^{sim}-B_{SL,k}^{exp}}{B_{SL,k}^{exp}}\right)}^{2}+\sum_{k=1}^{4}{\left(r\left(B_{Ca,k}^{sim},\left[B_{Ca,k}^{exp,lb},B_{Ca,k}^{exp,ub}\right]\right)\right)}^{2}\right]+Pen,$$

with

$$r=\left\{\begin{array}{cc} \;0, & if\;\;B_{Ca,k}^{sim}\in \left[B_{Ca,k}^{exp,lb},B_{Ca,k}^{exp,ub}\right]\\ \frac{B_{Ca,k}^{sim}-B_{Ca,k}^{exp,lb}}{B_{Ca,k}^{exp,lb}} & if\;\;B_{Ca,k}^{sim}<B_{Ca,k}^{exp,lb}\\ \frac{B_{Ca,k}^{sim}-B_{Ca,k}^{exp,ub}}{B_{Ca,k}^{exp,ub}} & if\;\;B_{Ca,k}^{sim}>B_{Ca,k}^{exp,ub} \end{array}\right.,$$

where $B_{SL,k}$ are sarcomere length biomarkers and $B_{Ca,k}$ are intracellular [Ca^2+^] biomarkers. The relative distance between simulated $\left(sim\right)$ and experimental $\left(exp\right)$ biomarker values was used because of different units. Experimental sarcomere length biomarkers were considered as median values while experimental intracellular [Ca^2+^] biomarkers were considered as a range with lower bound $\left(lb\right)$ defined as mean minus standard deviation and upper bound $\left(ub\right)$ defined as mean plus standard deviation. This was done to consider that intracellular [Ca^2+^] biomarker values were collected in different experiments with different subjects.

The minimisation problem was constrained to ensure that the parameters and resulting transients remained within a physiological range. Parameter constraints were of the form $p^{lb}\leq p\leq p^{ub}$, where lower and upper bounds were set to 50 % and 150 % of the reference value, respectively (Table S3). The reference value is the original model value from the literature except for certain parameters of the rat cardiomyocyte model, where the original model values were adjusted to ensure that the resulting range of parameters produces more transients without abnormalities. Transient constraints were formulated as penalty terms Pen in line with the abnormalities given in Section 2.1:

$$\begin{array}{l} Pen=\sum_{l=1}^{6}Pen^{l}=c_{1}\left[{\left(\frac{\min\left(SLAmp\right)}{\max\left(SLAmp\right)}-1\right)}^{2}+{\left(\frac{\max\left(CaAmp\right)}{\max\left(CaAmp\right)}-1\right)}^{2}\right]+c_{2}\left[{\left(max\left(0,max{\left.\left(\frac{dSL}{dt}\right)\right|}_{t<t_{peak}}\right)\right)}^{2}\right.\\ \left.\;\;\;\;\;\;\;\;\;\;\;\;\;\;\;\;\;\;\;\;\;\;\;\;\;\;\;\;\;\;\;\;\;+{\left(min\left(0,min{\left.\left(\frac{dSL}{dt}\right)\right|}_{t\geq t_{peak}}\right)\right)}^{2}+{\left(min\left(0,min{\left.\left(\frac{d{\left[Ca^{2+}\right]}_{i}}{dt}\right)\right|}_{t<t_{peak}}\right)\right)}^{2}+{\left(max\left(0,max{\left.\left(\frac{d{\left[Ca^{2+}\right]}_{i}}{dt}\right)\right|}_{t\geq t_{peak}}\right)\right)}^{2}\right], \end{array}$$

with the penalty coefficients $c=\left\{10^{2},10^{6}\right\}$. While $Pen^{1}$ and $Pen^{2}$ ensured the absence of alternans, $Pen^{3}-Pen^{6}$ ensured the absence of fluctuations. The penalty coefficients were set such that reaching any abnormality threshold leads to Pen≥1.

The population-based differential evolution method implemented in the Python package *LMFIT* (version 1.0.2) (Newville, Stensitzki, Allen, & Ingargiola, 2014) was used to solve the minimisation problem. Introduced by Storn and Price (1997), this meta-heuristic global optimisation method is used for multi-dimensional real-valued functions and does not require them to be differentiable and continuous, making it suitable for real-world problems. However, it should be noted that there is no guarantee that the global minimum is found. The default optimisation parameters of the *LMFIT* implementation were used and the optimisation was run only once for model recalibration as the aim was only to find one possible set of model parameters that can reproduce experimental data.

#### 2.4.4. Computation of Ca^2+^ sensitivity

The Ca^2+^ sensitivity represents the intracellular Ca^2+^ concentration that is required to produce half-maximal isometric active stress. Having steady-state active stresses computed for given intracellular Ca^2+^ concentrations, the Ca^2+^ sensitivity *Ca*_50_ can be determined using the Hill equation:

$$S_{a}=\frac{S_{a_{max}}}{1+{\left(\frac{pCa_{50}}{pCa}\right)}^{h_{Ca}}},$$

where pCa is defined as $6-\log_{10}\left({\left[Ca^{2+}\right]}_{i}\;\;\text{in}\;\;\mu \text{M}\right)$, $pCa_{50}$ is correspondingly defined as $6-\log_{10}\left(Ca_{50}\;\;\text{in}\;\;\mu \text{M}\right)$, $S_{a_{max}}$ is the maximum steady-state isometric active stress, and $h_{Ca}$ is the Hill coefficient. The data for pCa was generated between 5 and 7 in intervals of 0.25 and the Python package *Hillfit* (version 0.1.7; https://github.com/himoto/hillfit) was used to find $pCa_{50}$.

#### 2.4.5. Model evaluation

The recalibrated computer models were evaluated for their representation of physiological and pharmacological behaviour based on comparing simulated and experimental data. The evaluation of physiological behaviour started with a quantitative comparison between simulated and experimental baseline action potential duration biomarkers (Table S4). This was followed by a qualitative comparison of simulated and experimental pacing frequency relationships (0.5–2 Hz) for sarcomere length and intracellular [Ca^2+^] biomarkers related to inotropy and lusitropy. In the simulated data, a relationship was classified as positive or negative if the biomarker values increased or decreased, respectively. In the experimental data from rat cardiomyocytes (Fig. S2), a relationship was considered positive or negative if at least two statistically significant increases or decreases were observed between biomarker values at two frequencies, respectively, with no significant opposite difference in the remaining frequency pair. If neither a positive nor a negative relationship was found, it was classified as flat. Experimental data from human cardiomyocytes were obtained from the literature, and the authors’ assessments were followed.

The evaluation of pharmacological behaviour was performed using data from drugs considered in the Comprehensive in vitro Proarrhythmia Assay (CiPA) initiative (Colatsky et al., 2016), for which Hill equation parameters (Table S5) were available as inputs, and data on inotropic and lusitropic drug effects (Table S6) were available for comparison between simulations and experiments. It should be noted that some of the drugs have additional targets not accounted for in the models. Consistent with Longobardi, Sher, and Niederer (2021), subcellular effects on hERG, KvLQT1/mink, and Nav1.5-late were ignored in the rat computer model because the corresponding ion currents are not represented. The evaluation of the rat cardiomyocyte model involved qualitative comparisons between simulated and experimental drug effects. Experimental data were collected in various experiments with variable numbers of drug concentrations and comparisons were made solely for the top concentration. The experimental drug effect was classified as positive or negative if it was statistically significantly positive or negative, respectively, while the simulated drug effect was classified as positive or negative if the effect was ≥5 % or ≤ −5 %, respectively. The evaluation of the human cardiomyocyte model involved both qualitative and quantitative comparisons between simulated and experimental drug effects. Experimental data were obtained from a single study with four drug concentrations per drug and qualitative comparisons were made for all concentrations. Following Nguyen et al. (2017) and Abi-Gerges et al. (2020), both the simulated and experimental drug effect were classified as positive or negative if the effect (mean effect in experimental data) was ≥10 % or ≤ −25 %, respectively. If neither a positive nor a negative drug effect was found, the drug effect was classified as absent. Further qualitative comparisons in human cardiomyocytes were based on the drug concentration that causes half-maximal reduction of percentage sarcomere shortening (IC50^Short^). Experimental IC50^Short^ values are available and the simulated IC50^Short^ values were determined using the Python package *Hillfit* (version 0.1.7). It was assumed that drugs with an effect of ≤ −25 % on percentage sarcomere shortening would eventually lead to complete contractility inhibition, in line with the experimental analysis (Nguyen et al., 2017). Following Nguyen et al. (2017), both simulated and experimental drug effect were classified as negative if IC50^Short^ was equal to or smaller than the top drug concentration; otherwise, the drug effect was classified as absent (experimental study did not include positive inotropic drugs). Quantitative comparisons in human cardiomyocytes were performed for IC50^Short^ values for all drugs where simulated and experimental qualitative drug effects agreed. These comparisons were based on the absolute percentage error.

### 2.5. Global sensitivity analyses

Sobol’ global sensitivity analyses (Sobol”, 2001) were performed to compute the sensitivities of given outputs (sarcomere length and intracellular [Ca^2+^] biomarkers) to given inputs (either parameters of the model components or pharmacological scaling factors) to inform the parameter selection for the model recalibration and to identify differences in inotropic and lusitropic drug effects in rat and human cardiomyocytes. Input samples were generated using Saltelli’s sampling scheme (Saltelli, 2002), and the corresponding outputs were obtained from simulations.

To inform parameter selection for recalibration, the analysis considered 27 model parameters, resulting in 57,344 input samples $\left(n=1024\right)$ within the recalibration ranges (Table S3), and 11 biomarkers (Fig. S1). To identify differences in inotropic and lusitropic drug effects, the analysis included 8 pharmacological scaling factors associated with the proteins of interest represented in both the rat and human cardiomyocyte models (Table 1) and the 14 characteristic sarcomere length and intracellular [Ca^2+^] biomarkers. The number of input samples was 18,432 $\left(n=1024\right)$, chosen within a range of 0.6 and 1.0 to encompass a common inhibition range (Crumb, Vicente, Johannesen, & Strauss, 2016) while minimising abnormal transients or incomplete biomarker determinations. For each parameter sample, sarcomere length and intracellular [Ca^2+^] transients were simulated, and biomarkers were extracted. Transients exhibiting abnormalities or missing biomarker values were excluded from the analysis.

Saltelli’s method (Homma & Saltelli, 1996; Saltelli, 2002) was used to compute the total-effect sensitivity index for each input-output combination, accounting for input interactions. Since biomarker values are required for each parameter sample, missing values from excluded transients were assigned the mean of the existing biomarker values. A total-effect sensitivity index of zero indicates no sensitivity, while higher values indicate higher sensitivity. To enable comparison across combinations, the index was normalised $\left(S_{T}^{n}\right)$ as in Strocchi et al. (2023), with values approaching one indicating higher sensitivity. A threshold of 0.1 was used to define insensitivity. The Python package *SALib* (version 1.3.12) (Herman & Usher, 2017) was used for the sampling and computing the total-effect indices.

### 2.6. Computational drug effect translation

#### 2.6.1. Concept and assumptions

**Fig. 2.**
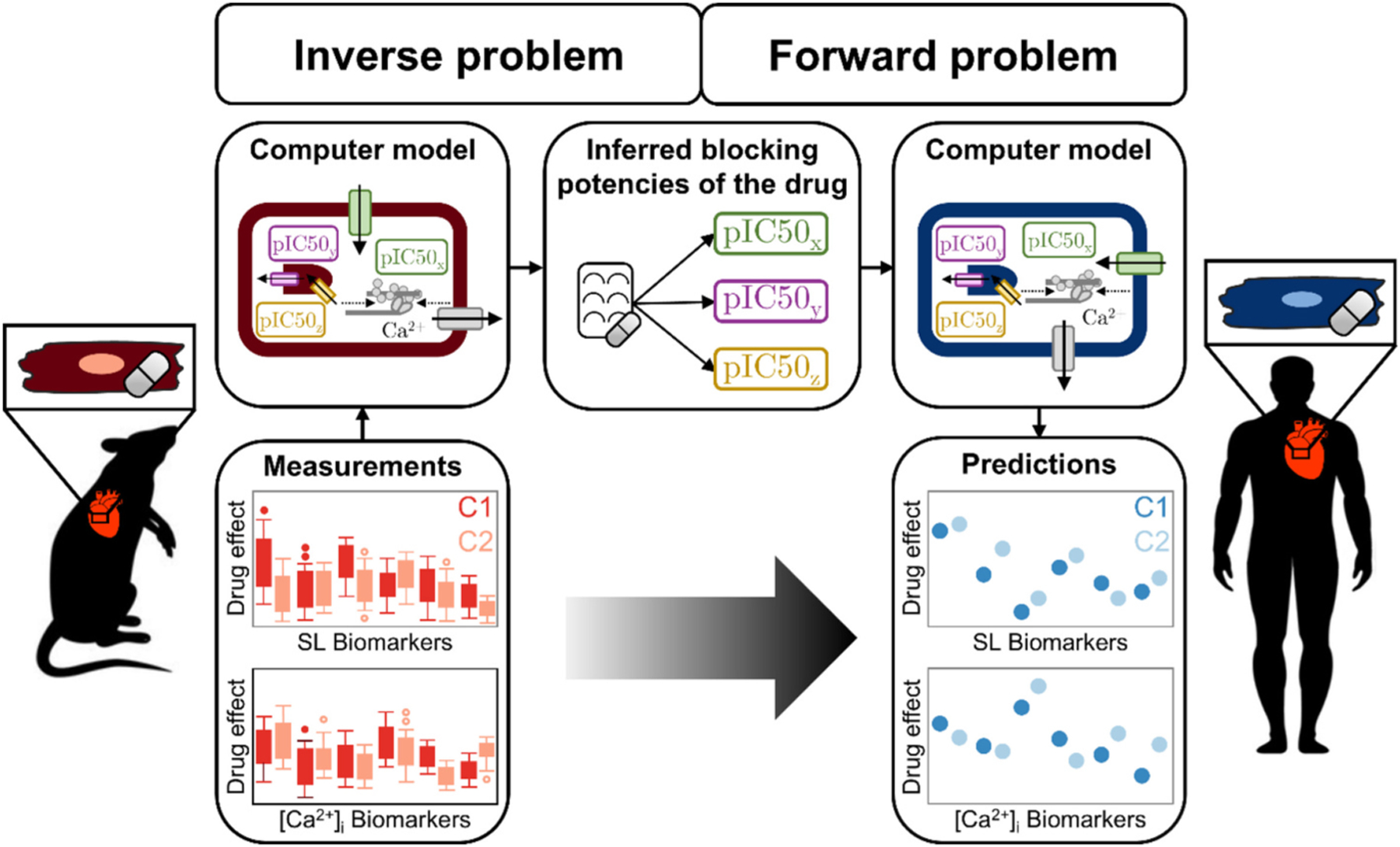
Concept of the computational drug effect translation approach that predicts human drug effects on sarcomere length and intracellular [Ca^2+^] biomarkers at multiple concentrations (C) based on measured rat drug effects.

The concept of computational drug effect translation (Fig. 2) builds on the work of Tveito et al. (2020), with the following foundational assumptions: 1) the proteins of interest are present in both rat and human ventricular cardiomyocytes (and are represented in the respective computer models), albeit potentially at different expression levels, and 2) drugs affect these proteins in the same manner.

#### 2.6.2. First step: Measurement of rat drug effects

The first step involves measuring the drug effects on the 14 characteristic sarcomere length and intracellular [Ca^2+^] biomarkers in rat cardiomyocytes at multiple concentrations. To this end, transients are collected before and after administering the given drug. Transients showing abnormalities are excluded from the analysis to facilitate the inverse problem in the second step (Grandits et al., 2024). The remaining transients are used to determine the biomarker values and corresponding drug effects. Since sarcomere length and intracellular [Ca^2+^] biomarkers are collected in different cardiomyocytes, medians across all included cardiomyocytes are computed and used as inputs for the second step.

#### 2.6.3. Second step: Inference of drug’s blocking potencies on proteins of interest

The second step involves using the rat cardiomyocyte model to infer the drug’s blocking potencies on the proteins of interest from the measured median drug effects (an inverse problem). To simplify the inference, we assume the Hill coefficient to be one (Mirams, 2023):

$$s_{p}=\frac{1}{1+\frac{C}{IC50_{p}}},$$

and introduce

$$pIC50_{p}=6-\log_{10}\left(IC50_{p}\;\;\text{in}\;\;\mu \text{M}\right)$$

as the parameter representing the drug’s blocking potency for the given protein p to obtain

$$s_{p}=\frac{1}{1+\frac{C}{10^{6-pIC50_{p}}}}.$$

In contrast to $IC50_{p}$, $pIC50_{p}$ ranges from −∞ to ∞, with larger values indicating stronger blocking potency. The advantages of this parameter transform are outlined in Whittaker, Clerx, Lei, Christini, and Mirams (2020).

The inverse problem is to find the blocking potencies that minimise the following objective function:

$$\sum_{j}\left[\frac{1}{14}\sum_{k}{\left(\Delta B_{k,j}^{exp}-\Delta B_{k,j}^{sim}\right)}^{2}+Pen_{j}\right],$$

where $\Delta B_{k,j}^{exp}$ are median experimental biomarker drug effects and $\Delta B_{k,j}^{sim}$ are simulated biomarker drug effects at the drug concentration j. The minimisation problem was constrained to ensure that the blocking potencies remained within a plausible range and that the resulting transients remained within a physiological range. Parameter constraints were of the form $p^{lb}\leq p\leq p^{ub}$, where lower and upper bounds were set to $6-log_{10}\left(C_{1}/100\right)$ and $6-log_{10}\left(100C_{4}\right)$ assuming that the $IC50_{p}$ is not lower than one-hundredth of the low concentration C_1_ and not higher than one hundred times the top concentration C_4_ similar to Johnstone, Bardenet, Gavaghan, and Mirams (2017). Transient constraints were formulated as penalty terms $Pen_{j}$ in line with the definitions of abnormalities given in Section 2.1. The penalty terms for the transients at each concentration were

$$\begin{array}{l} Pen_{j}=c_{1}\left[{\left(\frac{\min\left(SLAmp_{j}\right)}{\max\left(SLAmp_{j}\right)}-1\right)}^{2}+{\left(\frac{\max\left(CaAmp_{j}\right)}{\min\left(CaAmp_{j}\right)}-1\right)}^{2}\right]+c_{2}\left[{\left(max\left(0,max{\left.\left(\frac{dSL_{j}}{dt}\right)\right|}_{t<t_{peak}}\right)\right)}^{2}\right.+{\left(min\left(0,min{\left.\left(\frac{dSL_{j}}{dt}\right)\right|}_{t\geq t_{peak}}\right)\right)}^{2}\\ \left.\;\;\;\;\;\;\;\;\;\;\;\;\;\;\;\;\;\;\;+{\left(min\left(0,min{\left.\left(\frac{d{\left[Ca^{2+}\right]}_{i,j}}{dt}\right)\right|}_{t<t_{peak}}\right)\right)}^{2}+{\left(max\left(0,max{\left.\left(\frac{d{\left[Ca^{2+}\right]}_{i,j}}{dt}\right)\right|}_{t\geq t_{peak}}\right)\right)}^{2}\right], \end{array}$$

with the penalty coefficients $c=\left\{10^{4},10^{8}\right\}$. The penalty coefficients were set such that reaching any abnormality threshold leads to $Pen_{j}\geq 10^{2}$.

To solve the minimisation problem, the differential evolution method was used with default optimisation parameters, similar to the approach used in model recalibration (Section 2.4.3). However, finding the global minimum was crucial in this case. Therefore, for each data set, the optimisation was run ten times with different initial populations, created by the default Latin hypercube sampling method. The blocking potencies corresponding to the minimum objective were then chosen.

Following the assumption that drugs affect proteins in rat (r) and human (h) cardiomyocytes in the same manner, i.e.


$$I_{p}^{r,drug}=I_{p}^{r,base}\frac{1}{1+\frac{C}{10^{6-pIC50_{p}^{r}}}}$$

and

$$I_{p}^{h,drug}=I_{p}^{h,base}\frac{1}{1+\frac{C}{10^{6-pIC50_{p}^{h}}}}$$

with

$$pIC50_{p}^{r}=pIC50_{p}^{h}=pIC50_{p},$$

the inferred blocking potencies are used as inputs for the human cardiomyocyte model in the third step. Additionally, knowing the blocking potencies of the given proteins provides mechanistic insight into subcellular drug action.

#### 2.6.4. Third step: Prediction of human drug effects

The third step involves using the human cardiomyocyte model to predict drug effects on the 14 characteristic sarcomere length and intracellular [Ca^2+^] biomarkers in human cardiomyocytes at multiple concentrations based on the inferred blocking potencies (a forward problem). The drug concentrations may exceed those used in rat measurements to explore conditions that could amplify or diminish the inotropic and lusitropic responses to the drug or induce abnormalities.

#### 2.6.5. Identifiability analysis

The success of the computational drug effect translation relies on the accurate inference of the blocking potencies of drugs in the second step. Therefore, for all eight proteins of interest represented in both the rat and the human cardiomyocyte model (Table 1), an identifiability analysis of the associated blocking potencies was conducted to find the proteins for which accurate blocking potency inference is attainable. To this end, ten synthetic rat drug effect data sets were analysed, generated using virtual drugs administered to the rat cardiomyocyte model. These virtual drugs were formulated through random sets of blocking potencies (pIC50 within 5 to 6) and administered at a concentration of 1 μM. This corresponds to pharmacological scaling factors $s_{p}$ within 0.5 to 0.91, covering a common range of inhibition (Crumb et al., 2016). The drug effects on the characteristic biomarkers were collected from simulations of sarcomere length and intracellular [Ca^2+^] transients before and after drug administration. To be included in the dataset, the transients produced by a given drug had to exhibit no abnormalities, and biomarker drug effects had to remain within the common range of −100 % to 500 % (Abi-Gerges et al., 2020; Nguyen et al., 2017). The datasets were then used to infer the blocking potencies in line with the second step, and errors were quantified using the mean absolute error (MAE). Proteins for which blocking potencies were inferred with an MAE ≤ 0.1 were considered suitable for computational drug effect translation.

#### 2.6.6. Evaluation on synthetic data

First, the computational drug effect translation was evaluated on synthetic data. One hundred synthetic rat and human drug effect data sets were generated by one hundred virtual drugs that were administered at four concentrations to the rat and human cardiomyocyte models. Virtual drugs were formulated through random sets of blocking potencies (pIC50 within 5 to 6) for each protein that was considered suitable for computational drug effect translation, and these were administered at four concentrations (0.01, 0.1, 1, and 10 μM). This corresponds to pharmacological scaling factors $s_{p}$ within 0.09 to 0.999, covering a wide range of inhibition. The drug effects on the characteristic biomarkers were collected from simulations of sarcomere length and intracellular [Ca^2+^] transients before and after administration of each of the four concentrations. To be included in the dataset, the transients produced by a given drug had to exhibit no abnormalities, and biomarker drug effects had to remain within the common range of −100 % to 500 % (Abi-Gerges et al., 2020; Nguyen et al., 2017). Quantitative comparisons were made between the synthetic rat and human drug effects, as well as between the predicted and synthetic human drug effects for all biomarkers and drug concentrations. Errors of inferred blocking potencies were quantified using the MAE and the coefficient of determination (R^2^) provided an indication of how well the synthetic rat drug effects and the predicted human drug effects fit the synthetic human drug effects.

#### 2.6.7. Evaluation on experimental data

Second, the computational drug effect translation was evaluated on experimental data obtained after the administration of four concentrations of thapsigargin (Table S7). Thapsigargin was chosen because it is a potent and selective inhibitor of SERCA (Lytton, Westlin, & Hanley, 1991; Treiman, Caspersen, & Christensen, 1998), which is among the proteins for which blocking potencies are identifiable, and because experimental data from human cardiomyocytes are also available (Abi-Gerges et al., 2020). Comparisons were made between experimental rat and human drug effects, as well was between predicted and experimental human drug effects. Qualitative comparisons were made for drug effects on sarcomere length biomarkers. Following Nguyen et al. (2017) and Abi-Gerges et al. (2020), drug effects (mean effects in experimental data) were classified as positive or negative if the effect was ≥10 % or ≤ −25 %, respectively. If neither a positive nor a negative drug effect was found, the drug effect was classified as absent. Quantitative comparisons were made for the drug effect on the percentage sarcomere shortening. First, the actual drug effects were compared and second, the IC50^Short^ values were compared. Experimental human drug effects on the percentage sarcomere shortening were extracted from the respective figure of the publication using *WebPlotDigitizer* (version 4.7; https://apps.automeris.io/wpd/). The IC50^Short^ was computed in line with the model evaluation (Section 2.4.5) and for experimental drug effects, computations were performed based on the mean effects. To understand possible discrepancies between predicted and experimental human drug effects, the evaluation was supplemented by a study on synthetic data generated by administering a virtual SERCA2 blocker (pIC50=4.704, found in the same way as the other virtual drugs, but with the bounds $6-log_{10}C_{1}$ and $6-log_{10}C_{4}$.) at the same concentrations.

## 3. Results

### 3.1. Model recalibration

**Fig. 3.**
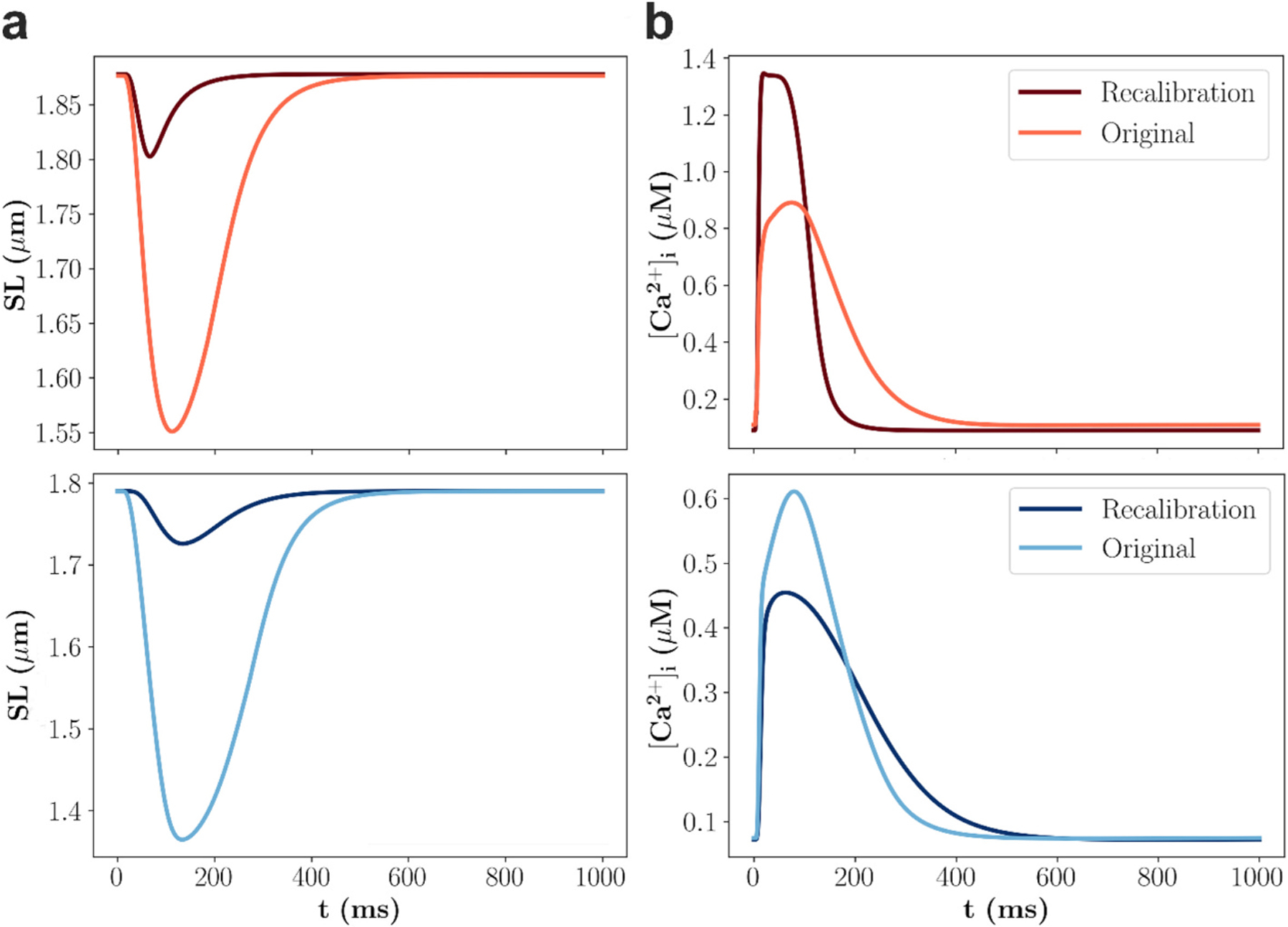
Simulated transients before and after recalibration of the rat and human cardiomyocyte models. (**a**) Sarcomere length and (**b**) intracellular [Ca^2+^] transients are shown, with transients from the rat and human cardiomyocyte modelsindicated in red and blue colour, respectively. (For interpretation of the references to colour in this figure legend, the reader is referred to the web version of this article.)

The recalibrated rat and human cardiomyocyte models produced sarcomere length biomarker values that all fall within experimental minima and maxima (Table 2). For intracellular calcium biomarkers, three out of four simulated values for the rat model were within the experimental means ± standard deviations, whereas none of the human model values fell within this range. However, deviations from experimental ranges were generally small. The largest absolute percentage errors were 5 % in the rat model (CaTP) and 44 % in the human model (for CaRes), with errors under 4 % for the other three human biomarkers. Notably, the amplitudes of the sarcomere length and intracellular [Ca^2+^] changed markedly after recalibration, but while the amplitude of the intracellular Ca^2+^ transient increased in the rat cardiomyocyte model and decreased in the human cardiomyocyte model, the amplitude of the sarcomere length transient decreased in both models (Fig. 3). This discrepancy can be attributed to a substantial reduction in Ca^2+^ sensitivity (Section 2.4.4) in the recalibrated rat cardiomyocyte model (from 0.6 to 1.6 μM), whereas the reduction in the recalibrated human cardiomyocyte model was comparably small (from 0.6 to 0.7 μM).

### 3.2. Model evaluation

**Fig. 4.**
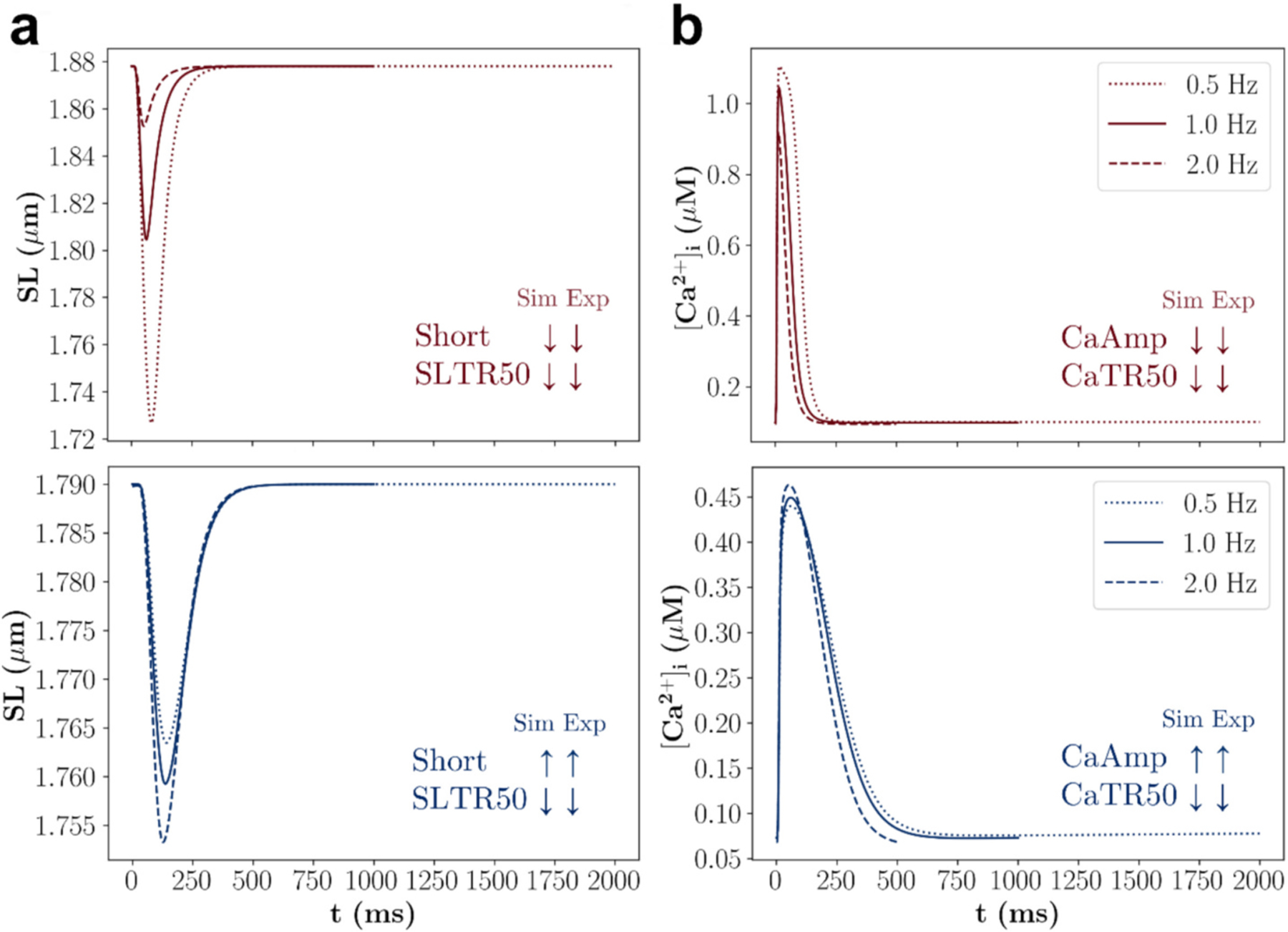
Evaluation of the rat and human cardiomyocyte models for their representation of physiological behaviour. (**a**) Sarcomere length and (**b**) intracellular [Ca^2+^] transients simulated at the pacing frequencies 0.5, 1, and 2 Hz are shown, and simulated and experimental pacing frequency relationships of biomarkers related to inotropy and lusitropy are compared. Red and blue colour indicate results of the rat and human cardiomyocyte model, respectively. Upwards and downwards arrows indicate positive and negative relationships, respectively. Note that the extracellular ion concentrations in the simulations with the human cardiomyocyte model were not adapted to match the experiments due to incomplete data availability. (For interpretation of the references to colour in this figure legend, the reader is referred to the web version of this article.)

First, the computer models were evaluated for their physiological behaviour. The rat and the human cardiomyocyte model produced simulated values of APD50 and APD90 that fall within the experimental ranges (Table 3). Furthermore, the simulated and experimental pacing frequency relationships (0.5 -to 2 Hz) agreed for all investigated sarcomere length and intracellular [Ca^2+^] biomarkers. Notably, the experimental pacing frequency relationships for Short and CaAmp were negative in rat cardiomyocytes and positive in human cardiomyocytes, and this important physiological difference was accurately reproduced by the computer models (Fig. 4).

Second, the computer models were evaluated for their pharmacological behaviour. The comparison of qualitative drug effects revealed that the rat cardiomyocyte model reproduced 80 % of the experimental inotropic drug effects and 50 % of the experimental lusitropic drug effects (Table 4). Using thresholds for drug effect classification (Abi-Gerges et al., 2020; Nguyen et al., 2017), the human cardiomyocyte model reproduced 52 % (low concentration), 70 % (2nd concentration), 61 % (3rd concentration) and 83 % (top concentration) of the experimental inotropic drug effects and 87 % (low concentration), 70 % (2nd concentration), 57 % (3rd concentration), and 57 % (top concentration) of the experimental lusitropic drug effects at four investigated concentrations. Furthermore, using the half-maximal reduction of Short (IC50^Short^) for drug effect classification (Nguyen et al., 2017), 87 % of the experimental inotropic drug effects were correctly determined. Of the three drugs where simulated and experimental drug effects did not agree, two (cisapride and droperidol) showed negative inotropic effects in the experiments. In the simulations, these effects were initially positive but, as further research indicated, shifted to negative at concentrations exceeding the experimental top concentration. For the 18 drugs where inotropic effects were classified identically in both experiments and simulations, the comparison of quantitative drug effects based on IC50^Short^ revealed a median (minimum, maximum) absolute percentage error of 64 (11,210) %.

### 3.3. Global sensitivity analyses

**Fig. 5.**
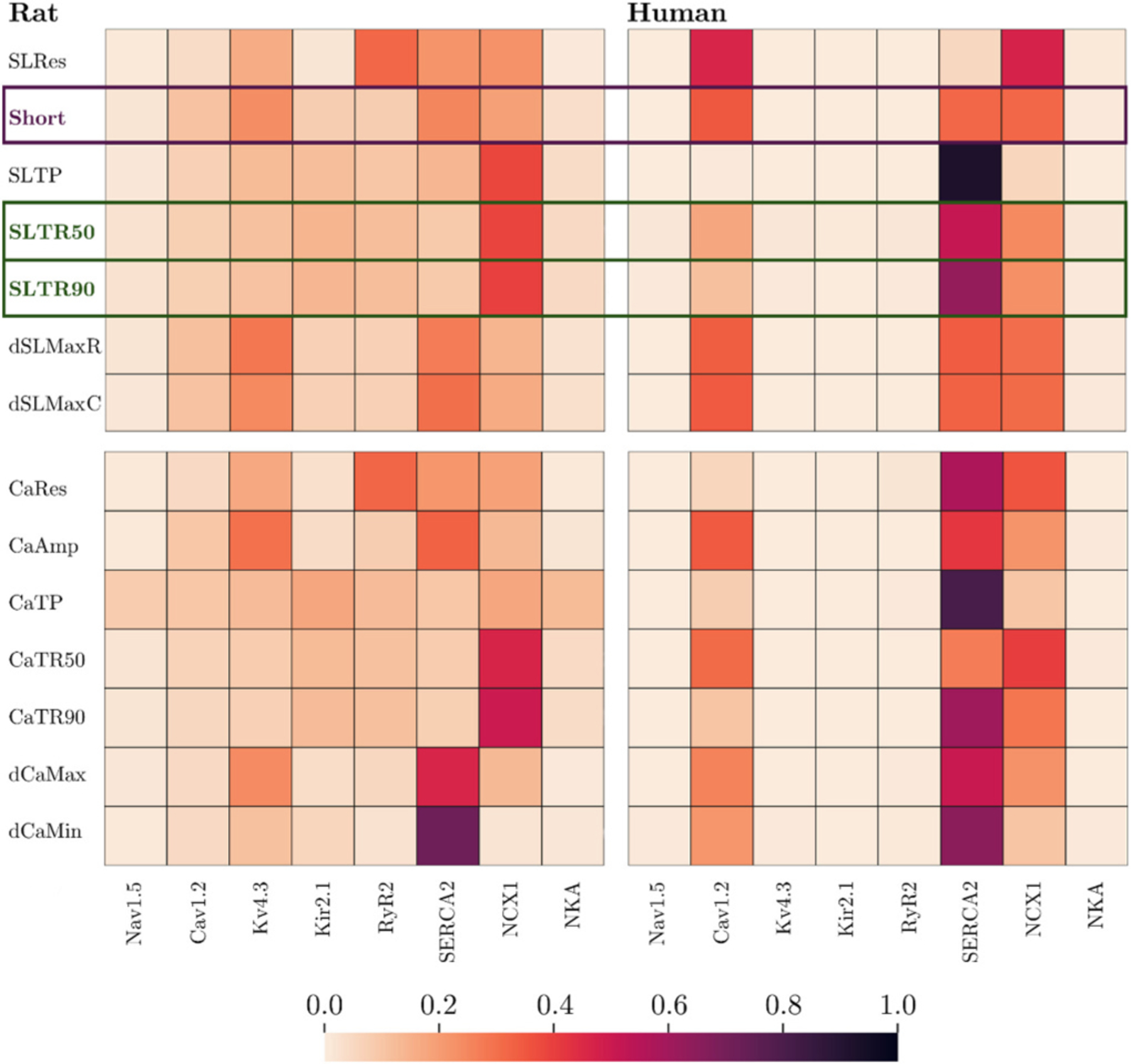
Global sensitivity analyses to identify different inotropic and lusitropic responses to inhibition of ion channels and transporters. Normalised total-effect sensitivity indices are shown and higher sensitivity is indicated by darker colour. The chosen biomarkers of inotropy and lusitropy are indicated in bold with framing, with inotropy markers in purple and lusitropy markers in green. The proportion of excluded transients was 2 % and 0 % in the analyses on the rat and human cardiomyocyte model, respectively. (For interpretation of the references to colour in this figure legend, the reader is referred to the web version of this article.)

Fig. 5 illustrates normalised total-effect sensitivities for each combination of proteins and biomarkers obtained from global sensitivity analyses. The analyses show that, in rat cardiomyocytes, targeting SERCA2, Kv4.3, NCX1, and Cav1.2 is effective $\left(S_{T}^{n}\geq 0.1\right)$ for modifying inotropy, while targeting NCX1, Kir2.1, RyR2, and Kv4.3 is effective for modifying lusitropy. In human cardiomyocytes, targeting Cav1.2, SERCA2, and NCX1 is effective for modifying both inotropy and lusitropy. The differences for inotropy and lusitropy are in line with differences for the amplitude and the return times of corresponding intracellular [Ca^2+^] transients.

### 3.4. Identifiability analysis

**Fig. 6.**
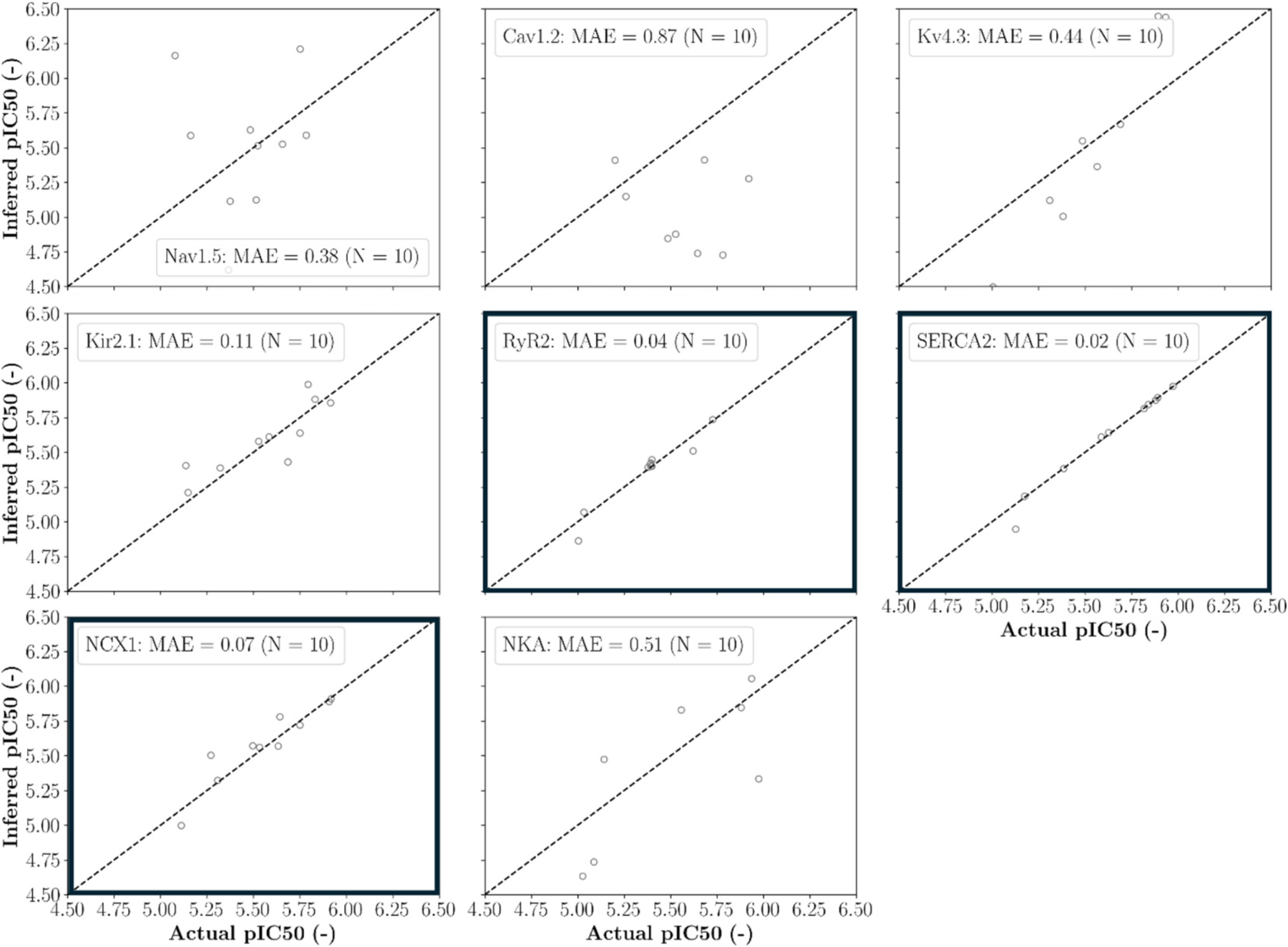
Identifiability analysis to inform the selection of proteins considered in the computational drug effect translation. Scatter plots show inferred versus actual blocking potencies and associated mean absolute errors (MAE). Bold frames indicate proteins considered in the computational drug effect translation (MAE ≤ 0.1).

The identifiability analysis revealed that the drug’s blocking potencies for RyR2, SERCA2, and NCX1 can be inferred from the characteristic sarcomere length and intracellular [Ca^2+^] biomarker values with an MAE below 0.1 (Fig. 6). These proteins were considered suitable for computational drug effect translation while the five other proteins were excluded.

### 3.5. Evaluation of the computational drug effect translation on synthetic data

**Fig. 7.**
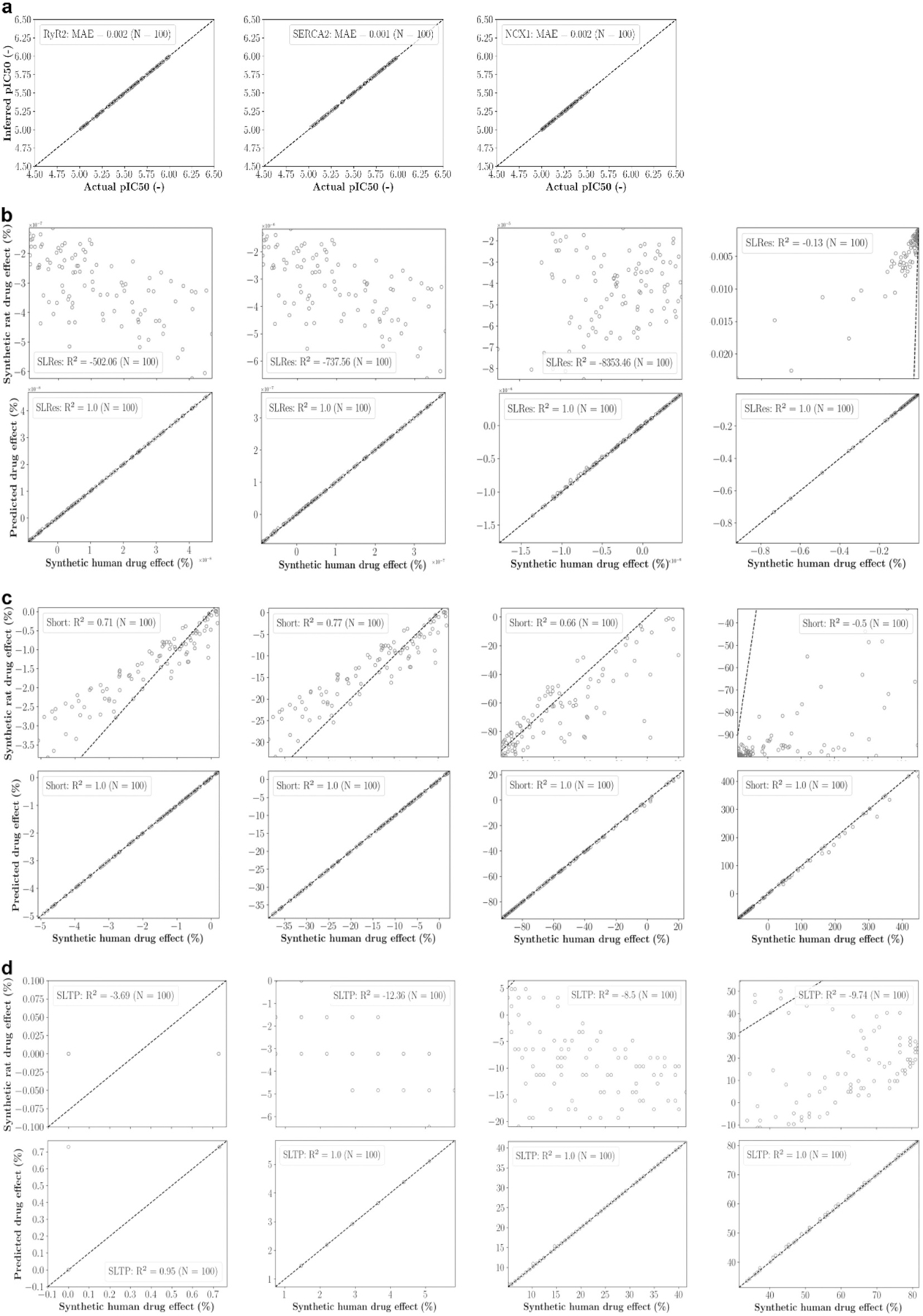

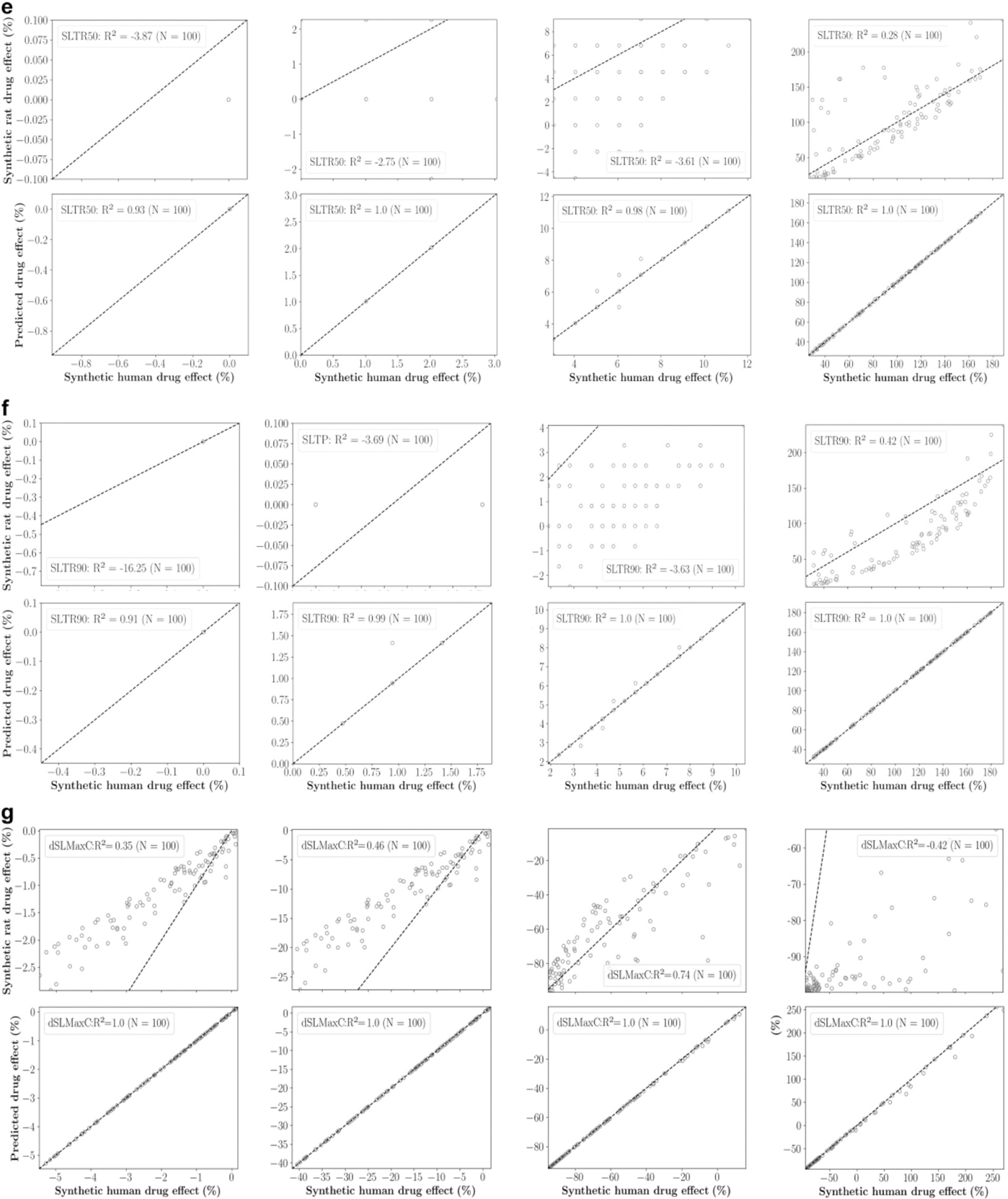

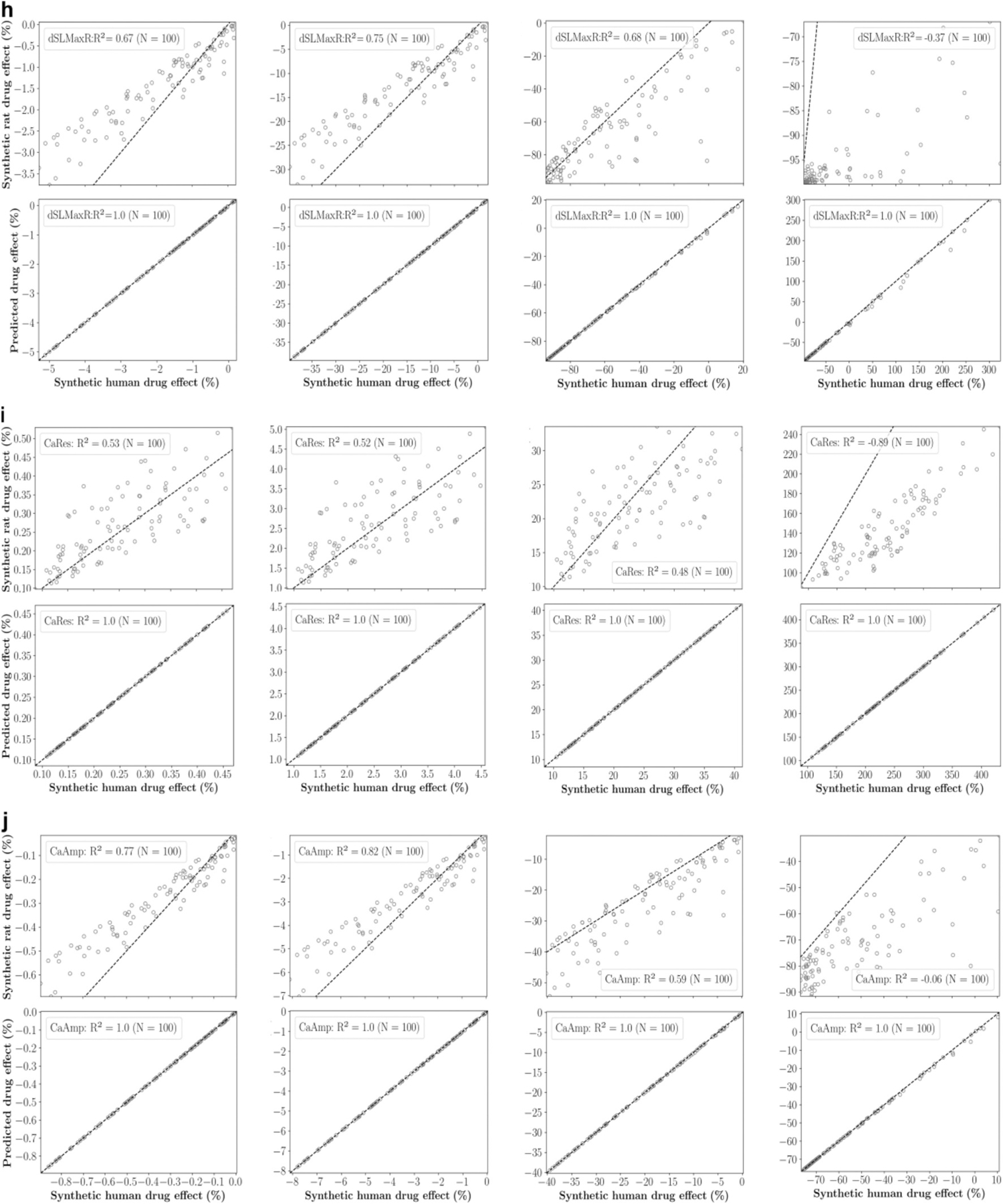

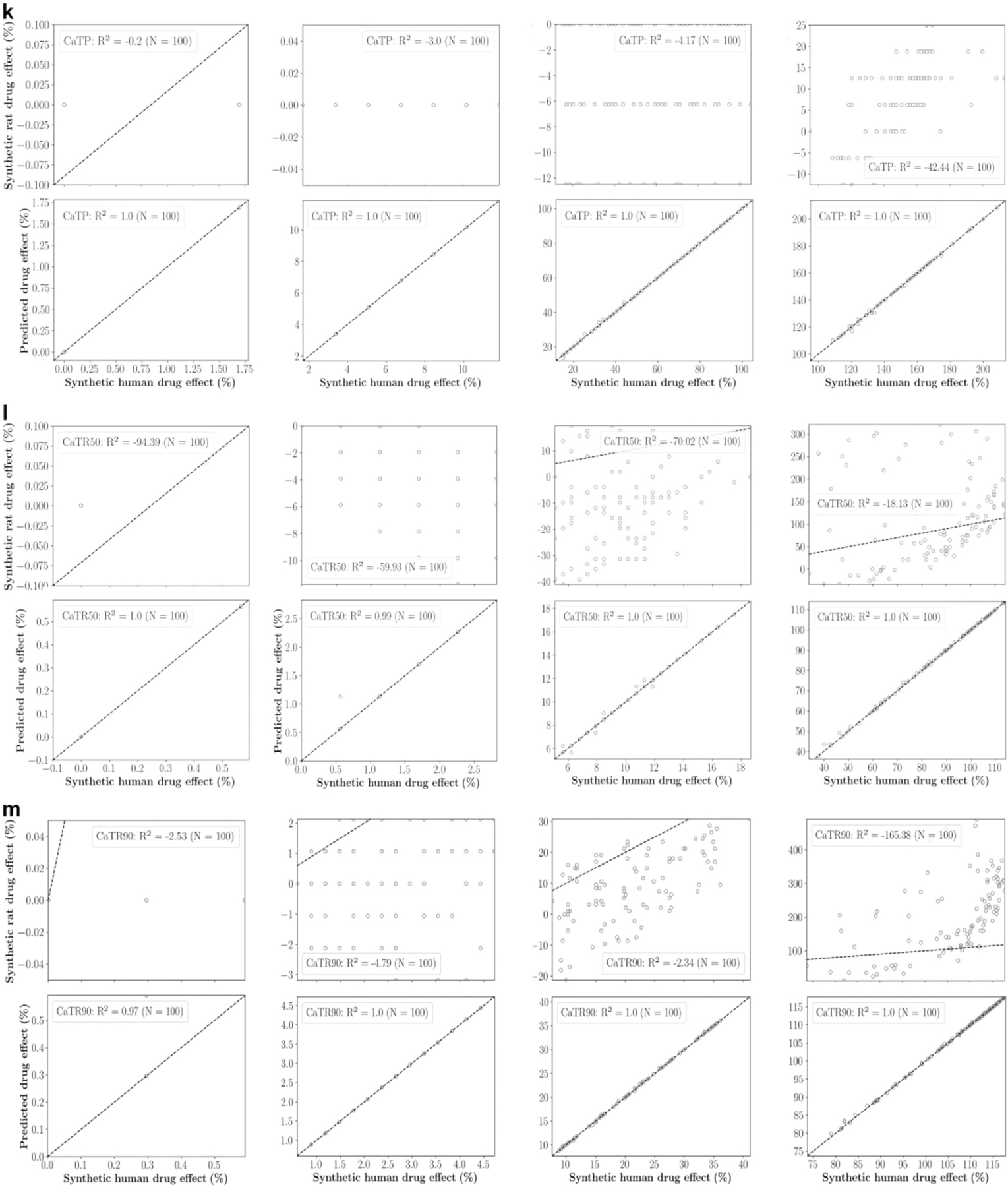

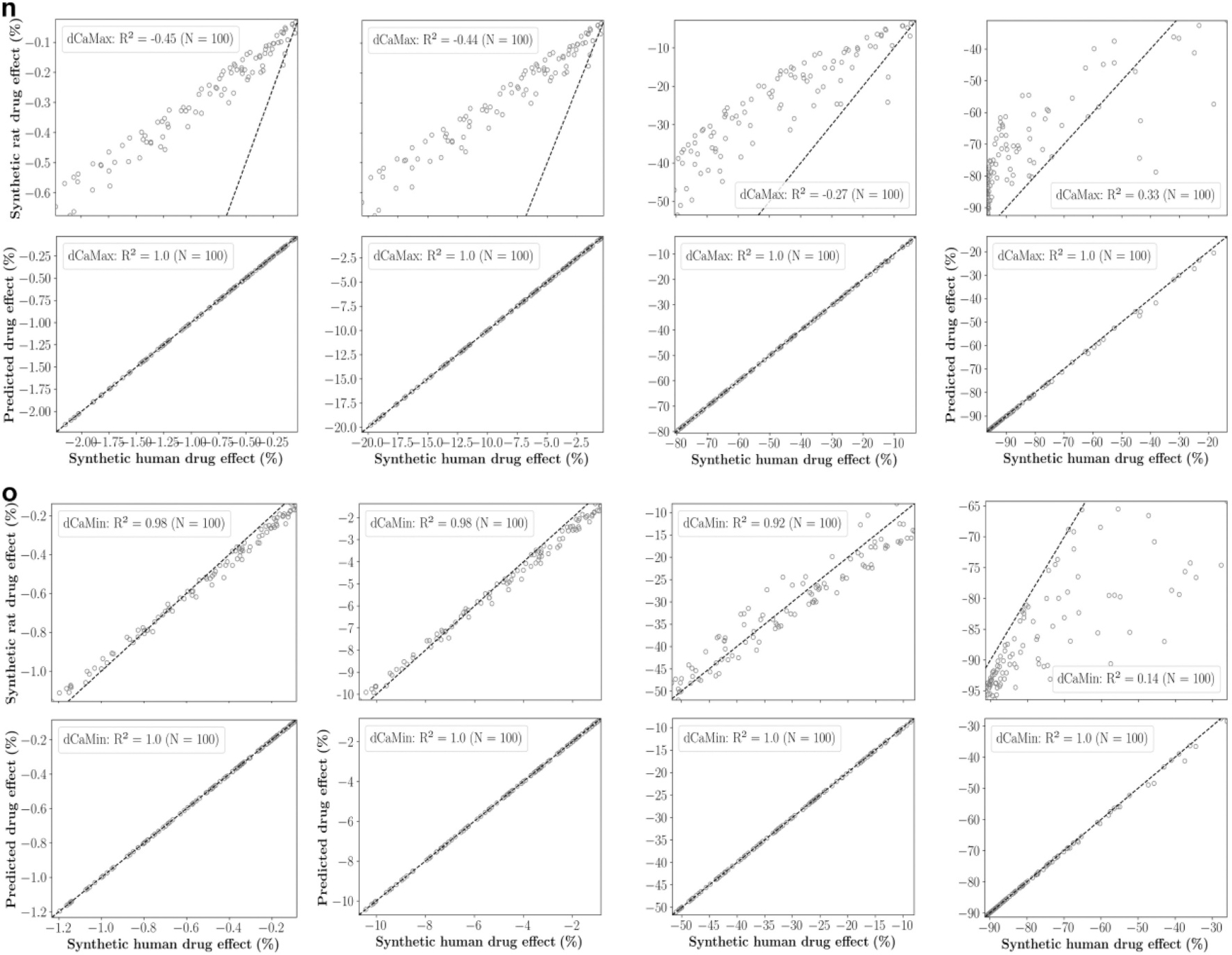
Evaluation of computational drug effect translation using synthetic data. (**a**) Scatter plots of inferred versus actual blocking potencies for RyR2, SERCA2, and NCX1, with mean absolute errors (MAE) provided. (**b**) - (**o**) Scatter plots of synthetic rat drug effects (upper panels) and predicted human drug effects (lower panels) versus synthetic human drug effects on the characteristic biomarkers. Results for increasing drug concentrations (0.01, 0.1, 1, and 10 μM) are shown from left to right, with coefficients of determination (R^2^) provided.

First, the computational drug effect translation was evaluated on one hundred synthetic data sets that were generated from virtual drugs targeting RyR2, SERCA2, and NCX1. The blocking potencies were inferred with MAEs of 0.002 (RyR2), 0.001 (SERCA2), and 0.002 (NCX1) (Fig. 7a). The R^2^ values of predicted human drug effects relative to synthetic human drug effects were much larger than those of rat drug effects relative to synthetic human drug effects across all biomarkers and concentrations (Table 5). Furthermore, R^2^ values between 0.9 and 1.0 (91 % were ≥ 0.99) indicate that the human drug effect predictions are precise, and the computational approach could also overcome different drug effect directions between rat and human cardiomyocytes (Fig. 7b–o).

### 3.6. Evaluation of the computational drug effect translation on experimental data

**Fig. 8.**
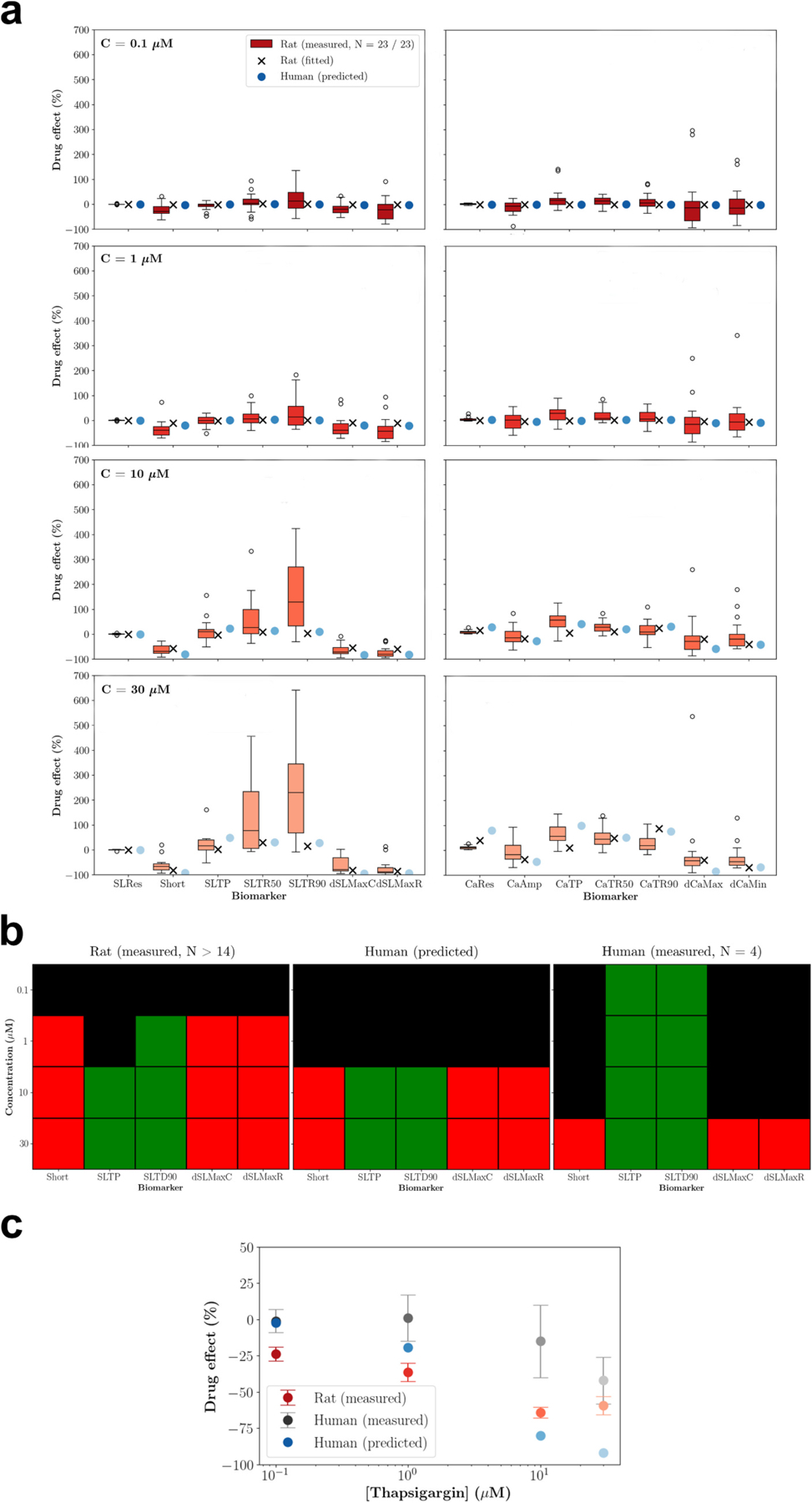
Evaluation of computational drug effect translation using experimental data collected for thapsigargin at four concentrations. (**a**) Experimental rat drug effects, corresponding fitting results, and predicted human drug effects. (**b**) Qualitative comparison of experimental rat drug effects, predicted human drug effects, and experimental human drug effects. Green, red, and black colours indicate positive (≥ 10 %), negative (≤ −25 %), and absent (> −25 % & < 10 %) drug effects, in line with the classification used in Nguyen et al. (2017) and Abi-Gerges et al. (2020). (**c**) Quantitative comparison of experimental rat drug effects, predicted human drug effects, and experimental human drug effects on the percentage sarcomere shortening. Experimental results are presented as means ± standard error of means. Details and sources of the experimental data can be found in Table S7. (For interpretation of the references to colour in this figure legend, the reader is referred to the web version of this article.)

Second, the computational drug effect translation was evaluated on experimental data obtained for thapsigargin at four concentrations. The inferred blocking potencies were 2.626 (RyR2), 4.725 (SERCA2), and 4.248 (NCX1). While the fitted rat drug effects during inference generally aligned well with the measurements, discrepancies emerged in CaRes at the top concentration, where the fitted drug effects exceeded the experimental observations (Fig. 8a). Comparisons of qualitative drug effects across the concentrations show that predicted human drug effects were more consistent with experimental human drug effects than with experimental rat drug effects for Short, dSLMaxC, and dSLMaxR but not for SLTP and SLTD90 (Fig. 8b). Comparisons of quantitative drug effects on Short across the concentrations show that the predicted human drug effects for the low and second concentration were not only closer to the experimental human drug effect than the experimental rat drug effects but also fell within the central 5 % and 47 % of the sampling distribution for the mean experimental data, respectively. This was not true for the third and top concentration (Fig. 8c). Moreover, the IC50^Short^ of rat measurements (4.2 μM) was slightly closer to human measurements (> 30 μM) than the human predictions (3.3 μM). However, further research using a virtual SERCA2 blocker showed qualitative and quantitative agreement between predicted and synthetic human drug effects across all biomarkers and concentrations (Fig. S3). The inferred blocking potencies were 4.703 (SERCA2; virtual drug: 4.704), 2.797 (RyR2), and 2.523 (NCX1).

## 4. Discussion

### 4.1. Translating inotropic and lusitropic drug effects from rats to humans using computer modelling

Global sensitivity analyses identified marked differences in inotropic and lusitropic responses to the inhibition of Cav1.2, Kv4.3, Kir2.1, RyR2, SERCA2, and NCX1. These are attributable to different electrophysiology and Ca^2+^ handling properties, primarily caused by variations in protein expression levels (Milani-Nejad & Janssen, 2014; Stevenson-Cocks, 2019), and explain inaccurate drug effect translations found in one hundred synthetic rat and human cardiomyocyte data sets that were generated from virtual drugs targeting RyR2, SERCA2, and NCX1. To bridge the gap, a computational drug effect translation approach was introduced and the evaluation on the same hundred synthetic data sets showed substantial improvement across all biomarkers and drug concentrations. The evaluation on experimental data obtained for thapsigargin also showed improvement, although not across all biomarkers and drug concentrations. Thapsigargin is a potent and selective SERCA inhibitor (Lytton et al., 1991; Treiman et al., 1998), as correctly indicated by the inferred blocking potencies being largest for SERCA2. However, the inferred blocking potency for NCX1 deviated by only −10 %, which contrasts the specificity of SERCA inhibition (Lytton et al., 1991; Treiman et al., 1998). To understand the discrepancy, we also performed the computational drug effect translation on synthetic data generated by the cardiomyocyte models with a virtual SERCA2 blocker. The inferred blocking potency for SERCA2 deviated from the virtual drug value by only −0.02 %, while deviations for RyR2 and NCX1 were − 41 % and − 46 %, respectively. This resulted in an excellent match between predicted and synthetic human drug effects, suggesting that the discrepancies in the experimental evaluation largely stem from disregarding intercellular variations of measured drug effects. These variations are likely a result of different underlying conductances of the proteins among the examined cardiomyocytes (Lachaud et al., 2022; Stokke, Louch, & Smith, 2024).

### 4.2. Limitations

#### 4.2.1. Experiments

First, measurements in rat cardiomyocytes could not be conducted at physiological pacing frequencies. Achieving such conditions in vitro may require using small tissue samples instead of isolated cardiomyocytes. Additionally, the ratiometric dye fura-2, used for measurements of intracellular [Ca^2+^], is known for intracellular [Ca^2+^] buffering (Takahashi, Camacho, Lechleiter, & Herman, 1999), and inhibition of the Na^+^/K^+^ ATPase (Smith et al., 2018) and the acto-myosin ATPase (Robinson et al., 2023). This results not only in distorted intracellular [Ca^2+^] measurements but, particularly due to the latter, in negative inotropic effects (Robinson et al., 2023). Therefore, sarcomere length and intracellular [Ca^2+^] were measured separately in different cardiomyocytes, impeding the consideration of intercellular variations of measured drug effects in the computational drug effect translation. Meanwhile, progress has been made in the optical measurement of intracellular [Ca^2+^], with genetically encoded Ca^2+^ indicators emerging as an alternative that addresses the aforementioned limitations of fura-2 (Robinson et al., 2023).

#### 4.2.2. Cardiomyocyte computer models

Inotropic and lusitropic drug effects can be modulated by Ca^2+^/calmodulin-dependent protein kinase II (CaMKII) signaling (Anderson, Brown, & Bers, 2011; Daniels et al., 2018; Gaido et al., 2023; Sossalla et al., 2010; Sutanto et al., 2020) and by β-adrenergic receptor signaling (Bristow, 2000; Lohse, Engelhardt, & Eschenhagen, 2003; Heijman, Volders, Westra, & Rudy, 2011; Ladage, Schwinger, & Brixius, 2013; Sutanto et al., 2020). However, while CaMKII signaling is already represented in the human cardiomyocyte model, it is lacking in the rat cardiomyocyte model (since the data-driven 1 Hz variant of the Gattoni model is used) and β-adrenergic receptor signaling is lacking in both models. Furthermore, mechano-electrical feedback mechanisms in both models were limited to modifications of Ca^2+^ buffering of troponin C but more exist (Timmermann et al., 2017; Quinn & Kohl, 2021) and incorporation is promising for further model improvement.

The subcellular drug effect model using the Hill equation is mostly adequate but in fact an oversimplification that can lead to inaccuracies in the prediction of electrophysiological drug effects (Farm et al., 2023) and probably also in the prediction of inotropic and lusitropic drug effects. To account for the drug’s complex binding kinetics would require the use of Markov models (Amanfu & Saucerman, 2011; Brennan, Fink, & Rodriguez, 2009; Li et al., 2017). However, while more detailed models of subcellular drug effects will undoubtedly offer valuable mechanistic insight into mechanisms of drug action and potentially improve the translation of drug effects, this advancement comes at the expense of a considerably larger parameter space that needs to be considered during inference.

#### 4.2.3. Computational drug effect translation

Solving the inverse problem is crucial in computational drug effect translation, as inaccurately inferred blocking potencies could lead to considerable errors in human predictions, particularly when their impact on inotropy and lusitropy in humans is large. Therefore, an identifiability analysis was performed to focus on those proteins for which blocking potencies can be accurately inferred. This led to only the three proteins SERCA2, RyR2, and NCX1, which poses a limitation to the approach as it narrows the spectrum of drugs that can be studied. Further improvements could be achieved through the use of information-rich experimental protocols for data collection (Whittaker et al., 2020). We also emphasise that inferred blocking potencies can be complemented by potencies for other proteins that have been independently measured in expression systems.

The computational cost of solving the inverse problem is substantial and would increase further when using Markov models, as well as with the number of examined cardiomyocytes, if intercellular variations of measured drug effects were also be accounted for. This can be mitigated using emulators. Gaussian process emulators (Coveney et al., 2021; Longobardi et al., 2021; Strocchi et al., 2023) and neural network emulators (Grandits et al., 2024; Regazzoni, Salvador, Dedè, & Quarteroni, 2022) have become popular in recent years. Given that transient abnormalities play a substantial role in pharmacological investigations, the latter are preferred due to their superior ability to handle such complexities (Grandits et al., 2024).

### 4.3. Augmentation of the drug development pipeline

Screening of inotropic and lusitropic drug effects begins as early as the lead selection phase and given the substantial number of drug candidates in this phase, cell- and tissue-based experimental models are increasingly employed to align with the 3R principles and to achieve higher throughput at lower resource intensity (Guth et al., 2019). To avoid translation issues resulting from species differences, hiPSC-derived cardiomyocytes are now preferred (Guth et al., 2019; Ribeiro et al., 2019) but these exhibit large variabilities between and even within cell lines, and lack maturity – shortcomings that have not been fully resolved yet (Ribeiro et al., 2019). Human primary cardiomyocytes, in contrast, have demonstrated excellent agreement between preclinical and clinical inotropic effects across a diverse spectrum of positive and negative inotropes. While limited availability has long hindered their routine use, much progress has been made towards their large-scale utilisation (Abi-Gerges et al., 2020; Nguyen et al., 2017). However, despite recent advancements, animal models will remain essential in preclinical drug development because the complexity of an entire organism cannot yet be fully represented by alternative models. At this juncture, the presented computational drug effect translation offers a potentially powerful augmentation to the current drug development pipeline. It not only bridges species differences and facilitates mechanistic understanding of subcellular drug action but also adheres to the 3R principles in that it ultimately reduces the reliance on animal models, as more information can be gained from the given data. The success of the CiPA paradigm (Strauss, Wu, Li, Koerner, & Garnett, 2021) has demonstrated that the use of computer modelling in drug development is no longer a theoretical concept but can be a practical and effective enhancement to the established methodologies. Introduced here for rat left ventricular cardiomyocytes, the computational drug effect translation should next be extended across scales to the whole organ level. Notably, substantial progress has already been achieved in modelling the electromechanics at the whole heart level (Augustin et al., 2021; Strocchi et al., 2023) laying the groundwork for this progression. Furthermore, the range of potential targets could be expanded towards incorporation of sarcomeric proteins (Hwang & Sykes, 2015) leveraging biophysically more detailed computer models of the contraction-relaxation processes (Regazzoni, Dedè, & Quarteroni, 2020). Beyond this, the framework could be adapted to other animal models and would enable integration of disease conditions, age, and sex, thereby stream-lining preclinical analyses before drug candidates are exposed to the first humans. The importance of considering age and sex in drug efficacy and safety screenings has been highlighted in numerous studies (Dekker et al., 2021; Fogli Iseppe et al., 2021; Peirlinck, Sahli Costabal, & Kuhl, 2021; Tannenbaum, Day, & Alliance, 2017; Yang & Clancy, 2012).

## 5. Conclusion

Computer modelling was used to address the challenge of translating inotropic and lusitropic drug effects from rats to humans at the cellular scale. To bridge the existing translation gap, we integrated cardiomyocyte models into a computational translation approach that infers blocking potencies for proteins from drug effect measurements in rat ventricular cardiomyocytes to make predictions in human ventricular cardiomyocytes. Evaluations on synthetic and experimental data demonstrated that computer modelling can improve the translation of inotropic and lusitropic drug effects, underscoring its potential as a tool to augment the current drug development pipeline.

## Supplementary Material

Supplementary material 1

Supplementary material 2

Appendix A. Supplementary data

Supplementary data to this article can be found online at https://doi.org/10.1016/j.vascn.2025.107747.

## Figures and Tables

**Table 1:** Proteins of interest and associated ion currents in the rat and the human cardiomyocyte computer models. The mathematical representation of these ion currents, along with experimental data for their original calibration, are provided in the publications describing the electrophysiological and Ca^2+^ handling components of the rat (Gattoni et al., 2016) and human (Tomek et al., 2019, 2020) cardiomyocyte models.

Protein	Ion current	Rat	Human
Nav1.5	Peak Na^+^ current		x
	Late Na^+^ current	x	x
Cav1.2	L-Type Ca^2+^ current	x	x
Kv4.3	Transient outward K^+^ current	x	x
hERG	Rapid delayed rectifier K^+^ current		x
KvLQT1/minK	Slow delayed rectifier K^+^ current		x
Kir2.1	Inward rectifier K^+^ current	x	x
RyR2	Ryanodine receptor current	x	x
SERCA2	Ca^2+^ pump current	x	x
NCX1	Na^+^/Ca^2+^ exchanger current	x	x
NKA	Na^+^/K^+^ pump current	x	x

**Table 2:** Comparison of simulated and experimental biomarker values after the recalibration of the (**a**) rat and (**b**) human cardiomyocyte model. Experimental intracellular [Ca^2+^] biomarkers are given as means ± standard deviations. Experimental rat (data set #1) and human (Nguyen et al., 2017) sarcomere length biomarkers are given as median values, and minima and maxima are given in brackets. The biomarkers SLTD70 and SLTD90 are the times from stimulus to 50 % and 90 % return, respectively. Details and sources of the experimental data can be found in Table S2.

(a)			

Biomarker	Unit	Simulation	Experiment

CaRes	μM	0.090	0.105 ± 0.016
CaPeak	μM	1.346	2.000 ± 1.000
CaTP	ms	20	30 ± 9
CaTR50	ms	91	82 ± 15
SLRes	μm	1.878	1.878 (Min: 1.794, Max: 1.970)
Short	%	4.02	4.14 (Min: 1.00, Max: 15.07)
SLTP	ms	66	61 (Min: 41, Max: 97)
SLTR50	ms	44	44 (Min: 19, Max: 93)
SLTR90	ms	121	80 (Min: 40, Max: 191)
dSLMaxC	μm/ms	−2.52•10^−3^	−2.08•10^−3^ (Min: −6.03•10^−3^, Max: −0.56•10^−3^)
dSLMaxR	μm/ms	1.05•10^−3^	1.37•10^−3^ (Min: 0.29•10^−3^, Max: 7.27•10^−3^)

(b)			

Biomarker	Unit	Simulation	Experiment

CaRes	μM	0.073	0.14 ± 0.01
CaAmp	μM	0.382	0.35 ± 0.02
CaTP	ms	62	50 ± 10
CaTR90	ms	333	375 ± 40
SLRes	μm	1.790	1.790 (Min: 1.490, Max: 1.960)
Short	%	3.582	3.79 (Min: 1.82, Max: 12.40)
SLTP	ms	135	162 (Min: 101, Max: 341)
SLTD70	ms	269	251 (Min: 147, Max: 560)
SLTD90	ms	342	314 (Min: 189, Max: 693)
dSLMaxC	μm/ms	−1.00•10^−3^	−0.80•10^−3^ (Min: −2.21•10^−3^, Max: −0.29•10^−3^)
dSLMaxR	μm/ms	0.43•10^−3^	0.82•10^−3^ (Min: 0.26•10^−3^, Max: 2.99•10^−3^)

**Table 3:** Comparison of simulated and experimental action potential biomarker values for the evaluation of physiological behaviour in the (**a**) rat and (**b**) human cardiomyocyte model. Experimental biomarkers are given as means ± standard deviations and minima and maxima, respectively. The APD50 and APD90 are the action potential durations at 50 % and 90 % repolarisation, respectively, measured from the instant of the maximum depolarisation rate. Details and sources of the experimental data can be found in Table S4.

(a)			

Biomarker	Unit	Simulation	Experiment

APD50	ms	9	10.07 ± 1.72
APD90	ms	48	43.41 ± 7.08

(b)			

Biomarker	Unit	Simulation	Experiment

APD50	ms	200	Min: 106.6, Max: 349.4
APD90	ms	265	Min: 178.1, Max: 442.7

**Table 4:** Comparison of simulated and experimental biomarker drug effects for the evaluation of pharmacological behaviour in the (**a**) rat and (**b**) human cardiomyocyte model. Qualitative positive and negative drug effects are indicated by an upward and downward arrow, respectively, and absent drug effects are indicated by a straight line. The pacing frequency of the experiments in rat cardiomyocytes could not always be adopted in the simulations due to potential simulation failure. Instead, the closest possible pacing frequencies were used, and these are provided in (**a**) in brackets. The pharmacological behaviour in the human cardiomyocyte model was also evaluated using quantitative drug effects based on the concentration that causes half-maximal reduction of the percentage sarcomere shortening (IC50^Short^), provided in (**b**) in brackets. Details and sources of the experimental data can be found in Table S6.

(a)			

Drug	Biomarker	Simulation	Experiment

Diltiazem	Short	↓ (0.9 Hz)	↓ (0.5 Hz)
Nifedipine	Short	↓	↓
Ranolazine	Short	—	↓
	SLTR50	—	—
Tamoxifen	SLAmp	↓	↓
	SLTR50	↓ (0.75 Hz)	↑ (0.2 Hz)
Verapamil	Short	↓ (0.9 Hz)	↓ (0.5 Hz)

(b)			

Drug	Biomarker	Simulation	Experiment

Astemizole	Short	↑ ↑ ↑ ↑	— — — —
	SLTD90	— — — —	— — — —
	IC50^Short^	— (> 0.009 μM)	— (> 0.009 μM)
Azimilide	Short	— — ↓ ↓	— — ↓ ↓
	SLTD90	— — — —	— — — ↑
	IC50^Short^	↓ (1.331 μM)	↓ (1.07 μM)
Bepridil	Short	— — ↓ ↓	— — ↓ ↓
	SLTD90	— — — —	— ↑ ↑ —
	IC50^Short^	↓ (0.19 μM)	↓ (0.7 μM)
Chlorpromazine	Short	— — ↓ ↓	↑ — — ↓
	SLTD90	— — — —	— — — —
	IC50^Short^	↓ (0.628 μM)	↓ (1.02 μM)
Cisapride	Short	↑ ↑ ↑ ↑	↓ ↓ ↓ ↓
	SLTD90	— — — —	— ↑ ↑ ↑
	IC50^Short^	— (> 0.258 μM)	↓ (0.02 μM)
Clarithromycin	Short	— ↓ ↓ ↓	↓ ↓ ↓ ↓
	SLTD90	— — — ↑	↑ ↑ ↑ ↑
	IC50^Short^	↓ (3.2 μM)	↓ (16 μM)
Clozapine	Short	— — ↓ ↓	— — ↓ ↓
	SLTD90	— — — —	— — — ↑
	IC50^Short^	↓ (0.78 μM)	↓ (1.5 μM)
Diltiazem	Short	↓ ↓ ↓ ↓	— ↓ ↓ ↓
	SLTD90	— — — ↑	— — — ↑
	IC50^Short^	↓ (0.02 μM)	↓ (1 μM)
Dofetilide	Short	↑ ↑ ↓ ↓	— — — —
	SLTD90	— — — —	— ↑ ↑ ↑
	IC50^Short^	↓ (0.059 μM)	— (> 0.22 μM)
Domperidone	Short	↓ ↓ ↓ ↓	— ↓ ↓ ↓
	SLTD90	— — ↑ ↑	↑ ↑ ↑ ↑
	IC50^Short^	↓ (0.004 μM)	↓ (0.2 μM)
Droperidol	Short	↑ ↑ ↑ —	— — ↓ ↓
	SLTD90	— — — —	— — ↑ —
	IC50^Short^	— (> 0.48 μM)	↓ (0.18 μM)
Ibutilide	Short	↑ ↑ — ↓	↑ — ↓ ↓
	SLTD90	— — — —	↑ ↑ ↑ ↑
	IC50^Short^	↓ (2.9 μM)	↓ (2 μM)
Loratadine	Short	— — ↓ ↓	— — — ↓
	SLTD90	— — — —	— — — —
	IC50^Short^	↓ (0.01563 μM)	↓ (0.0175 μM)
Mexiletine	Short	— ↓ ↓ ↓	↓ ↓ ↓ ↓
	SLTD90	— — — —	— — — ↑
	IC50^Short^	↓ (2.79 μM)	↓ (0.9 μM)
Nifedipine	Short	↓ ↓ ↓ ↓	— ↓ ↓ ↓
	SLTD90	— — — —	— — — ↑
	IC50^Short^	↓ (0.002 μM)	↓ (0.04 μM)
Nitrendipine	Short	↓ ↓ ↓ ↓	— — ↓ ↓
	SLTD90	— — — —	— — — —
	IC50^Short^	↓ (0.0009 μM)	↓ (0.06 μM)
Ondansetron	Short	— — ↓ ↓	— — — ↓
	SLTD90	— — — —	— — — —
	IC50^Short^	↓ (2.5 μM)	↓ (14 μM)
Quinidine	Short	— ↓ ↓ ↓	— ↓ ↓ ↓
	SLTD90	— — — ↑	— ↑ ↑ ↑
	IC50^Short^	↓ (2.95 μM)	↓ (3.6 μM)
Ranolazine	Short	— ↓ ↓ ↓	— ↓ ↓ ↓
	SLTD90	— — — —	— — — ↑
	IC50^Short^	↓ (11.1 μM)	↓ (17 μM)
Sotalol	Short	— ↑ ↑ —	— ↑ ↑ —
	SLTD90	— — — —	— — ↑ ↑
	IC50^Short^	— (> 450 μM)	— (> 450 μM)
Tamoxifen	Short	— — ↓ ↓	— — — ↓
	SLTD90	— — — —	— — — —
	IC50^Short^	↓ (0.437 μM)	↓ (0.99 μM)
Vandetanib	Short	— ↓ ↓ ↓	— — ↓ ↓
	SLTD90	— — — —	— — ↑ ↑
	IC50^Short^	↓ (0.72 μM)	↓ (2.7 μM)
Verapamil	Short	— ↓ ↓ ↓	— ↓ ↓ ↓
	SLTD90	— — ↑ —	— — — —
	IC50^Short^	↓ (0.025 μM)	↓ (0.04 μM)

**Table 5:** Evaluation of computational drug effect translation based on synthetic data. The coefficients of determination (R^2^) are provided for (**a**) synthetic rat drug effects and (**b**) predicted human drug effects relative to synthetic human drug effects, across four concentrations (C_1–4_). The number of data sets was *N* = 100.

(a)

Biomarker	R^2^			
	C_1_	C_2_	C_3_	C_4_

SLRes	< 0	< 0	< 0	< 0
Short	0.709	0.771	0.658	< 0
SLTP	< 0	< 0	< 0	< 0
SLTR50	< 0	< 0	< 0	0.276
SLTR90	< 0	< 0	< 0	0.418
dSLMaxC	0.346	0.465	0.739	< 0
dSLMaxR	0.668	0.755	0.677	< 0
CaRes	0.526	0.516	0.475	< 0
CaAmp	0.774	0.822	0.590	< 0
CaTP	< 0	< 0	< 0	< 0
CaTR50	< 0	< 0	< 0	< 0
CaTR90	< 0	< 0	< 0	< 0
dCaMax	< 0	< 0	< 0	0.335
dCaMin	0.978	0.979	0.918	0.137

(**b**)

Biomarker	R^2^			
	C_1_	C_2_	C_3_	C_4_

SLRes	1.000	1.000	0.999	1.000
Short	1.000	1.000	1.000	0.997
SLTP	0.954	1.000	1.000	1.000
SLTR50	0.926	1.000	0.982	1.000
SLTR90	0.905	0.990	0.997	1.000
dSLMaxC	1.000	1.000	1.000	0.997
dSLMaxR	1.000	1.000	1.000	0.996
CaRes	1.000	1.000	1.000	1.000
CaAmp	1.000	1.000	1.000	0.999
CaTP	1.000	1.000	1.000	0.999
CaTR50	1.000	0.994	0.997	0.999
CaTR90	0.967	1.000	1.000	0.999
dCaMax	1.000	1.000	1.000	0.999
dCaMin	1.000	1.000	1.000	0.998

## Data Availability

All experimental data obtained in rat ventricular cardiomyocytes and all synthetic data generated for the evaluation of the computational drug effect translation are available in *Zenodo*: https://doi.org/10.5281/zenodo.15314866.

## Code availability

The cardiomyocyte computer models are included in the freely available software *openCARP*.
