# Supplementary material for: Computational modelling for improved translation of cardiac inotropic and lusitropic drug effects from rats to humans
Source: J Pharmacol Toxicol Methods. Author manuscript; Available in PMC 2026 Jan 3. (PMC12759087; doi:10.1016/j.vascn.2025.107747)
Supplement: Supplementary material 1 [file NIHMS2122390-supplement-Supplementary_material_1.docx]

**Supplementary Tables**

**Table S1.** Characteristics of the isolated rat left ventricular cardiomyocytes in all data sets. Strain, sex, age, body mass, heart mass, and number of isolated cardiomyocytes for the measurements of sarcomere length and intracellular [Ca^2+^] are given for each rat. Sprague Dawley and Wistar rats are abbreviated by CD and WI. The total number of cardiomyocytes N and the number of cardiomyocytes with included data (in brackets) is given below for (**a**) data set #1 (**b**) data set #2 (number of cardiomyocytes with included data is equal in all pacing frequencies since data exclusion applied to the entire set for the given cardiomyocyte), and (**c**) data set #3 (number of cardiomyocytes with included data refer separately to baseline and each of the four drug concentrations).

(**a**)

|  |  |  |  |  |  |  |  |
| --- | --- | --- | --- | --- | --- | --- | --- |
| **ID** | **Strain** | **Sex** | **Age (weeks)** | **Body mass**  **(g)** | **Heart mass (mg)** | **N^SL^** | **N^Ca^** |
|  |  |  |  |  |  |  |  |
|  |  |  |  |  |  |  |  |
| 64 | CD | f | 21 | 414 | 1464 | 6 | 2 |
| 71 | CD | m | 26 | 686 | 2217 | 5 | 5 |
| 72 | CD | f | 18 | 373 | 1438 | 5 | 4 |
| 75 | CD | m | 28 | 689 | 2335 | 17 | 13 |
|  |  |  |  |  |  |  |  |
|  |  |  |  |  |  | 33 (33) | 24 (24) |
|  |  |  |  |  |  |  |  |

(**b**)

|  |  |  |  |  |  |  |  |
| --- | --- | --- | --- | --- | --- | --- | --- |
| **ID** | **Strain** | **Sex** | **Age (weeks)** | **Body mass**  **(g)** | **Heart mass (mg)** | **N^SL^** | **N^Ca^** |
|  |  |  |  |  |  |  |  |
|  |  |  |  |  |  |  |  |
| 97 | CD | m | 44 | 635 | 2217 | 10 | 10 |
| 74 | CD | f | 38 | 401 | 1555 | 6 | 5 |
| 76 | CD | f | 43 | 647 | 1768 | 10 | 10 |
| 7731 | WI | m | 81 | 407 | 1958 | 5 | 5 |
| 117 | CD | m | 45 | 779 | 2498 | 5 | 0 |
|  |  |  |  |  |  |  |  |
|  |  |  |  |  |  | 36 (31) | 30 (28) |
|  |  |  |  |  |  |  |  |

(**c**)

|  |  |  |  |  |  |  |  |
| --- | --- | --- | --- | --- | --- | --- | --- |
| **ID** | **Strain** | **Sex** | **Age (weeks)** | **Body mass**  **(g)** | **Heart mass (mg)** | **N^SL^** | **N^Ca^** |
|  |  |  |  |  |  |  |  |
|  |  |  |  |  |  |  |  |
| 78 | CD | f | 41 | 391 | 1503 | 9 | 1 |
| 111 | CD | m | 50 | 670 | 2073 | 5 | 5 |
| 109 | CD | m | 51 | 707 | 2333 | 10 | 10 |
| 119 | CD | m | 46 | 720 | 2600 | 5 | 10 |
|  |  |  |  |  |  |  |  |
|  |  |  |  |  |  | 29  (23, 23, 22, 21, 14) | 26  (23, 23, 21, 17, 16) |
|  |  |  |  |  |  |  |  |

**Table S2.** Sources and details of the experimental data used for the recalibration of the (**a**) rat cardiomyocyte model and (**b**) human cardiomyocyte model. Sarcomere length and intracellular [Ca^2+^] biomarker data were obtained.

(**a**)

|  |  |
| --- | --- |
| **Data** | **Source and details** |
|  |  |
|  |  |
| Sarcomere length | Data set #1: data collected in left ventricular cardiomyocytes at 35-37°C and 1 Hz pacing (monophasic stimulation for 5 ms); extracellular ion concentrations: [Na^+^] = 140 mM, [Ca^2+^] = 1.5 mM, [K^+^] = 4 mM |
| Intracellular [Ca^2+^] | (Gattoni et al., 2016): data collected in left ventricular cardiomyocytes at 37°C and 1 Hz pacing (monophasic stimulation for 3 ms); extracellular ion concentrations: [Na^+^] = 140 mM, [Ca^2+^] = 1.8 mM, [K^+^] = 5.4 mM |
|  |  |

(**b**)

|  |  |
| --- | --- |
| **Data** | **Source and details** |
|  |  |
|  |  |
| Sarcomere length | (Nguyen et al., 2017): data collected in ventricular cardiomyocytes at 37°C and 1 Hz pacing (biphasic stimulation for 3 ms); extracellular ion concentrations: [Na^+^] = 145 mM, [Ca^2+^] = 1.8 mM, [K^+^] = 4 mM, [Cl^−^] = 154.6 mM |
| Intracellular [Ca^2+^] | (Coppini et al., 2013): data (adopted from Strocchi et al. (2023)) collected in septal cardiomyocytes at 35 ± 0.5°C and 1 Hz pacing (monophasic stimulation for 3 ms); extracellular ion concentrations: [Na^+^] = 132 mM, [Ca^2+^] = 1.8 mM, [K^+^] = 4 mM, [Cl^−^] = 142 mM |
|  |  |

**Table S3.** Rat and human cardiomyocyte model parameters considered for the Sobol’ global sensitivity analysis and recalibration. Their reference values, and their ranges for the Sobol’ global sensitivity analysis and recalibration are given. The recalibrated values are also provided. (**a**) Rat electrophysiology and Ca^2+^ handling model component (Gattoni model), (**b**) human electrophysiology and Ca^2+^ handling model component (ToR-ORd-dynCl model), and (**c**) contraction-relaxation model component (Land model). The reference values are the original values from literature except for some parameters of the rat cardiomyocyte model which were adjusted to ensure that the resulting range of values produces more transients without abnormalities (original model values from literature are given in brackets). Note that the reference value and range for ${[{Ca}^{2+}]}_{T50}^{ref}$ of the contraction-relaxation model component were adjusted for the rat cardiomyocyte model, while the original value and corresponding range were retained in the human cardiomyocyte model.

(**a**)

|  |  |  |  |  |  |
| --- | --- | --- | --- | --- | --- |
| **Parameter** | **Unit** | **Reference value** | **Range** | **Rat**  **value** | **Human value** |
|  |  |  |  |  |  |
|  |  |  |  |  |  |
| $g_{Na}$ | (mS) | 7.000∙10^−4^ | [3.500∙10^−4^, 10.500∙10^−4^] | - | - |
| $J_{L}$ | (µs^3^/ms) | 4.667∙10^−4^  (7.000∙10^−4^) | [2.334∙10^−4^, 7.000∙10^−4^] | 6.361∙10^−4^ | - |
| $g_{t}$ | (mS) | 3.920∙10^−5^  (1.960∙10^−5^) | [1.960∙10^−5^, 5.880∙10^−5^] | 2.497∙10^−5^ | - |
| $g_{K1}$ | (mS) | 4.000∙10^−5^ | [2.000∙10^−5^, 6.000∙10^−5^] | 3.501∙10^−5^ | - |
| $J_{R}$ | (µs^3^/ ms) | 2.000∙10^−2^ | [1.000∙10^−2^, 3.000∙10^−2^] | - | - |
| $g_{SERCA}$ | (mmol/Lms) | 4.700∙10^−4^ | [2.350∙10^−4^, 7.050∙10^−4^] | 7.050∙10^−4^ | - |
| $g_{NCX}$ | (mmol/Lms) | 10.300∙10^−2^  (5.150∙10^−2^) | [5.150∙10^−2^, 15.450∙10^−2^] | 6.832∙10^−2^ | - |
| $I_{NaK\_max}$ | (µA) | 1.380∙10^−3^ | [0.690∙10^−3^, 2.070∙10^−3^] | 1.457∙10^−3^ | - |
| ${\bar{\left[ {Ca}^{2+} \right]}}_{TRPN}$ | (mmol/L) | 7.000∙10^−2^ | [3.500∙10^−2^, 10.500∙10^−2^] | - | - |
|  |  |  |  |  |  |

(**b**)

|  |  |  |  |  |  |
| --- | --- | --- | --- | --- | --- |
| **Parameter** | **Unit** | **Reference**  **value** | **Range** | **Rat**  **value** | **Human value** |
|  |  |  |  |  |  |
|  |  |  |  |  |  |
| $G_{Na}$ | (mS/µF) | 1.178∙10^1^ | [0.589∙10^1^, 1.767∙10^1^] | - | - |
| $P_{{Ca}_{b}}$ | (-) | 8.376∙10^−5^ | [4.188∙10^−5^, 12.564∙10^−5^] | - | 5.07∙10^−5^ |
| $G_{{to}_{b}}$ | (mS/µF) | 1.600∙10^−1^ | [0.800∙10^−1^, 2.4∙10^−1^] | - | - |
| $G_{{K1}_{b}}$ | (mS/µF) | 6.992∙10^−1^ | [3.496∙10^−1^, 10.488∙10^−1^] | - | - |
| $J_{{rel}_{b}}$ | (-) | 1.5378∙10^0^ | [0.769∙10^0^, 2.307∙10^0^] | - | - |
| $J_{{up}_{b}}$ | (-) | 1.000∙10^0^ | [0.500∙10^0^, 1.500∙10^0^] | - | 0.593∙10^0^ |
| $G_{{ncx}_{b}}$ | (mS/µF) | 3.400∙10^−3^ | [1.700∙10^−3^, 5.100∙10^−3^] | - | 1.706∙10^−3^ |
| $P_{{NaK}_{b}}$ | (mS/µF) | 1.545∙10^1^ | [0.773∙10^1^, 2.318∙10^1^] | - | - |
| ${\bar{\left[ {Ca}^{2+} \right]}}_{TRPN}$ | (mmol/L) | 7.000∙10^−2^ | [3.500∙10^−2^, 10.500∙10^−2^] | - | 10.45∙10^−2^ |
|  |  |  |  |  |  |

(**c**)

|  |  |  |  |  |  |
| --- | --- | --- | --- | --- | --- |
| **Parameter** | **Unit** | **Reference value** | **Bounds** | **Rat**  **value** | **Human value** |
|  |  |  |  |  |  |
|  |  |  |  |  |  |
| $k_{TRPN}$ | (1/ms) | 1.000∙10^−1^ | [0.500∙10^−1^, 1.500∙10^−1^] | - | - |
| $n_{TRPN}$ | (-) | 2.000∙10^0^ | [1.000∙10^0^, 3.000∙10^0^] | 2.994∙10^0^ | 2.689∙10^0^ |
| ${[{Ca}^{2+}]}_{T50}^{ref}$ | (µM) | 1.600∙10^0^  (0.805∙10^0^) | [0.805∙10^0^, 2.415∙10^0^]  ([0.403∙10^0^, 1.208∙10^0^]) | 2.411∙10^0^ | 1.031∙10^0^ |
| $k_{u}$ | (1/ms) | 1.000∙10^0^ | [0.500∙10^0^, 1.500∙10^0^] | - | - |
| $n_{TM}$ | (-) | 5.000∙10^0^ | [2.500∙10^0^, 7.500∙10^0^] | 3.771∙10^0^ | 7.426∙10^0^ |
| ${TRPN}_{50}$ | (-) | 3.500∙10^−1^ | [1.750∙10^−1^, 5.250∙10^−1^] | 2.386∙10^−1^ | 3.407∙10^−1^ |
| $\nu$ | (-) | 7.000∙10^0^ | [3.500∙10^0^, 10.500∙10^0^] | - | - |
| $\mu$ | (-) | 3.000∙10^0^ | [1.500∙10^0^, 4.500∙10^0^] | - | - |
| $r_{W}$ | (-) | 5.000∙10^−1^ | [2.500∙10^−1^, 7.500∙10^−1^] | - | - |
| $r_{S}$ | (-) | 2.500∙10^−1^ | [1.250∙10^−1^, 3.750∙10^−1^] | 1.251∙10^−1^ | 1.904∙10^−1^ |
| $\gamma_{S}$ | (-) | 8.500∙10^−3^ | [4.250∙10^−3^, 12.750∙10^−3^] | - | - |
| $\gamma_{W}$ | (-) | 6.150∙10^−1^ | [3.075∙10^−1^, 9.925∙10^−1^] | - | - |
| $\phi$ | (-) | 2.230∙10^0^ | [1.115∙10^0^, 3.345∙10^0^] | - | - |
| $A_{eff}$ | (-) | 2.500∙10^1^ | [1.250∙10^1^, 3.750∙10^0^] | - | - |
| $\beta_{0}$ | (-) | 2.300∙10^0^ | [1.15∙10^0^, 3.45∙10^0^] | - | - |
| $\beta_{1}$ | (-) | −2.400∙10^0^ | [−3.6∙10^0^, −1.2∙10^0^] | - | - |
| $T_{ref}$ | (kPa) | 1.200∙10^2^ | [0.600∙10^2^, 0.800∙10^2^] | - | - |
| $a$ | (kPa) | 2.100∙10^0^ | [1.050∙10^0^, 3.150∙10^0^] | - | - |
|  |  |  |  |  |  |

**Table S4.** Sources and details of the experimental data used for the evaluation of the (**a**) rat and (**b**) human cardiomyocyte model. Action potential, sarcomere length, and intracellular [Ca^2+^] biomarker data were collected. The EGTA concentration is provided in the details of the action potential measurements in rat cardiomyocytes, as it was also considered in the corresponding simulations.

(**a**)

|  |  |
| --- | --- |
| **Data** | **Source and details** |
|  |  |
|  |  |
| Action potential | (Gattoni et al., 2016): data collected in left ventricular cardiomyocytes at 37°C and 1 Hz pacing (monophasic stimulation for 3 ms); extracellular ion concentrations: [Na^+^] = 140 mM, [Ca^2+^] = 1.8 mM, [K^+^] = 5.4 mM; [EGTA] = 0.06 mM |
| Pacing frequency relationship of sarcomere length | Data set #2: data collected in left ventricular cardiomyocytes at 35-37°C and 0.5 - 2 Hz pacing (monophasic stimulation for 5 ms); extracellular ion concentrations: [Na^+^] = 140 mM, [Ca^2+^] = 1.5 mM, [K^+^] = 4.0 mM |
|  | (Williams et al., 2014): data collected in ventricular cardiomyocytes at 34-36°C and 0.5 - 3 Hz pacing (monophasic stimulation for 3 ms); extracellular ion concentrations: [Na^+^] = 149.8 mM, [Ca^2+^] = 1.5 mM, [K^+^] = 5 mM |
| Pacing frequency relationship of intracellular [Ca^2+^] | Data set #2: data collected in left ventricular cardiomyocytes at 35-37°C and 0.5 - 2 Hz pacing (monophasic stimulation for 5 ms); extracellular ion concentrations: [Na^+^] = 140 mM, [Ca^2+^] = 1.5 mM, [K^+^] = 4.0 mM |
|  |  |

(**b**)

|  |  |
| --- | --- |
| **Data** | **Source and details** |
|  |  |
|  |  |
| Action potential | (Britton et al., 2017): data collected in right ventricular trabeculae and papillary tissue at 37°C and 1 Hz pacing (monophasic stimulation for 2 ms); extracellular ion concentrations (O’Hara et al., 2011): [Na^+^] = 144.33 mM, [Ca^2+^] = 1.8 mM, [K^+^] = 4.0 mM, [Cl^−^] = 152.66 mM |
| Pacing frequency relationship of sarcomere length | (Høydal et al., 2018): data collected in left ventricular cardiomyocytes at 22 ± 0.5°C and 0.5 - 2 Hz pacing. |
| Pacing frequency relationship of intracellular [Ca^2+^] | (Høydal et al., 2018): data collected in left ventricular cardiomyocytes at 22 ± 0.5°C and 0.5 - 2 Hz pacing. |
|  |  |

**Table S5.** Experimental drug parameter data used for the pharmacological evaluation of the rat and human cardiomyocyte model. The parameters are the half-maximal inhibitory concentration (${IC}_{50}$) and the Hill coefficient ($h$) given for various proteins (**Table 1**). If available, data published in Li et al. [2017] were used since more proteins are considered. Otherwise, data published in Li et al. [2019] were used, which consider only Nav1.5-peak, Nav1.5-late, Cav1.2, and hERG.

|  |  |  |  |  |  |  |  |
| --- | --- | --- | --- | --- | --- | --- | --- |
| **Drug** | Nav1.5-  peak | Nav1.5-late | Cav1.2 | Kv4.3 | hERG | KvLQT1/  minK | Kir2.1 |
|  |  |  |  |  |  |  |  |
|  |  |  |  |  |  |  |  |
| Astemizole  ^Li2019^ |  |  |  |  |  |  |  |
| IC50 (µM)  h | 5.41  0.76 | 10.3  2.3 | 0.553  1.2 | -  - | 0.00999  0.54 | -  - | -  - |
| Azimilide  ^Li2019^ |  |  |  |  |  |  |  |
| IC50 (µM)  h | 363  0.72 | 2940  0.47 | 13.2  0.71 | -  - | 0.828  0.75 | -  - | -  - |
| Bepridil  ^Li2017^ |  |  |  |  |  |  |  |
| IC50 (µM)  h | 2.9293  1.2 | 1.8139  1.4 | 2.8081  0.6 | 8.594  3.5 | 0.05  0.9 | 28.6283  0.7 | -  - |
| Chlorpromazine  ^Li2017^ |  |  |  |  |  |  |  |
| IC50 (µM)  h | 4.5356  2 | 4.5596  0.9 | 8.1919  0.8 | 17616.711  0.4 | 0.9292  0.8 | -  - | 9.2699  0.7 |
| Cisapride  ^Li2017^ |  |  |  |  |  |  |  |
| IC50 (µM)  h | -  - | -  - | 9258.076  0.4 | 219.1124  0.2 | 0.0101  0.7 | 81192.862  0.3 | 29.498  0.5 |
| Clarithromycin  ^Li2019^ |  |  |  |  |  |  |  |
| IC50 (µM)  h | 1090  0.89 | 1810  3 | 38.1  0.88 | -  - | 62.1  0.72 | -  - | -  - |
| Clozapine  ^Li2019^ |  |  |  |  |  |  |  |
| IC50 (µM)  h | 257  0.59 | 73.6  2 | 5.49  0.94 | -  - | 2.63  0.73 | -  - | -  - |
| Diltiazem  ^Li2017^ |  |  |  |  |  |  |  |
| IC50 (µM)  h | 110.859  0.7 | 21.8685  0.7 | 0.1121  0.7 | 2.82∙10^6^  0.2 | 13.15  0.9 | -  - | -  - |
| Dofetilide  ^Li2017^ |  |  |  |  |  |  |  |
| IC50 (µM)  h | 0.3805  0.9 | 753.1604  0.3 | 0.2603  1.2 | 0.0188  0.8 | 0.0049  0.9 | -  - | 0.3943  0.8 |
| Domperidone  ^Li2019^ |  |  |  |  |  |  |  |
| IC50 (µM)  h | 41.9  1.5 | 225  2.1 | 0.0736  0.49 | -  - | 0.132  0.78 | -  - | -  - |
| Droperidol  ^Li2019^ |  |  |  |  |  |  |  |
| IC50 (µM)  h | 36.6  2.5 | 33.8  2.8 | 3.23  1.2 | -  - | 0.0403  1.1 | -  - | -  - |
| Ibutilide  ^Li2019^ |  |  |  |  |  |  |  |
| IC50 (µM)  h | 24.1  2.3 | 287  2.4 | 37  0.86 | -  - | 0.0146  1.1 | -  - | -  - |
| Loratadine  ^Li2019^ |  |  |  |  |  |  |  |
| IC50 (µM)  h | 113  1.4 | 192  2.1 | 0.703  0.56 | -  - | 27.2  0.63 | -  - | -  - |
| Mexiletine  ^Li2017^ |  |  |  |  |  |  |  |
| IC50 (µM)  h | -  - | 8.9568  1.4 | 38.2436  1 | - | 28.88  0.9 | - | - |
| Nifedipine  ^Li2019^ |  |  |  |  |  |  |  |
| IC50 (µM)  h | 27.6  1.1 | 45.6  4.5 | 0.0114  0.67 | -  - | 142  0.75 | -  - | -  - |
| Ondansetron  ^Li2017^ |  |  |  |  |  |  |  |
| IC50 (µM)  h | 57.6664  1 | 19.1808  1 | 22.5514  0.8 | 1023.378  1 | 1.32  0.9 | 569.807  0.7 | -  - |
| Quinidine  ^Li2017^ |  |  |  |  |  |  |  |
| IC50 (µM)  h | 12.329  1.5 | 9.417  1.3 | 51.5923  0.6 | 3.4874  1.3 | 0.992  0.8 | 4.8989  1.4 | 39589.919  0.4 |
| Ranolazine  ^Li2017^ |  |  |  |  |  |  |  |
| IC50 (µM)  h | 68.774  1.4 | 7.8845  0.9 | - | - | 8.27  0.9 | 36155.02  0.5 |  |
| Sotalol  ^Li2017^ |  |  |  |  |  |  |  |
| IC50 (µM)  h | 1.14∙10^6^  0.5 | - | 7061.527  0.9 | 43143.455  0.7 | 110.6  0.8 | 4221.856  1.2 | 3050.26  1.2 |
| Tamoxifen  ^Li2019^ |  |  |  |  |  |  |  |
| IC50 (µM)  h | 84  0.82 | 3640  4 | 5.72  0.76 | - | 3.49  0.78 | - | - |
| Vandetanib  ^Li2019^ |  |  |  |  |  |  |  |
| IC50 (µM)  h | 80.9  1.9 | 3790  0.82 | 6.06  0.72 | - | 0.251  0.81 | - | - |
| Verapamil  ^Li2017^ |  |  |  |  |  |  |  |
| IC50 (µM)  h | 7.028  1 | 13.4292  0.8 | 0.2018  1.1 | - | 0.288  1 | - | 3.49∙10^5^  0.3 |
|  |  |  |  |  |  |  |  |

**Table S6.** Sources and details of the experimental biomarker data used for the pharmacological evaluation of the (**a**) rat and (**b**) human cardiomyocyte model. Sarcomere length biomarker drug effect data were collected.

(**a**)

|  |  |
| --- | --- |
| **Drug** | **Source and details** |
|  |  |
|  |  |
| Diltiazem | (Bell and McDermott, 1995): data collected in ventricular cardiomyocytes at 37°C and 0.5 Hz pacing (biphasic stimulation for 1 ms); drug concentrations: 10 µM; extracellular ion concentrations: [Na^+^] = 125 mM, [Ca^2+^] = 2.0 mM, [K^+^] = 5.8 mM |
| Nifedipine | (Bokenes et al., 2005): data collected in left ventricular cardiomyocytes at 37°C and 1 Hz pacing (biphasic stimulation for 5 ms); drug concentrations: 100 µM; extracellular ion concentrations: [Na^+^] = 140 mM, [Ca^2+^] = 1.8 mM, [K^+^] = 5.4 mM |
| Ranolazine | (Williams et al., 2014): data collected in ventricular cardiomyocytes at 34-36°C and 1 Hz pacing (monophasic stimulation for 3 ms); drug concentrations: 10 µM; extracellular ion concentrations: [Na^+^] = 149.8 mM, [Ca^2+^] = 1.5 mM, [K^+^] = 5 mM |
| Tamoxifen | (Asp al., 2013): data collected in ventricular cardiomyocytes at 36 ± 1°C and 0.2 Hz pacing (monophasic stimulation for 3 ms); drug concentrations: 10 µM; extracellular ion concentrations: [Na^+^] = 140 mM, [Ca^2+^] = 1.8 mM, [K^+^] = 5 mM |
| Verapamil | (Bell and McDermott, 1995): data collected in ventricular cardiomyocytes at 37°C and 0.5 Hz pacing (biphasic stimulation for 1 ms); drug concentrations: 10 µM; extracellular ion concentrations: [Na^+^] = 125 mM, [Ca^2+^] = 2.0 mM, [K^+^] = 5.8 mM |
|  |  |

(**b**)

|  |  |
| --- | --- |
| **Drug** | **Source and details** |
|  |  |
|  |  |
| Astemizole | (Nguyen, 2017): data collected in ventricular cardiomyocytes at 36°C and 1 Hz pacing (biphasic stimulation for 3 ms); drug concentrations 0.0003, 0.0009, 0.003, and 0.009 µM; extracellular ion concentrations: [Na^+^] = 145 mM, [Ca^2+^] = 1.8 mM, [K^+^] = 4 mM, [Cl^−^] = 154.6 mM |
| Azimilide | (Nguyen, 2017): data collected in ventricular cardiomyocytes at 36°C and 1 Hz pacing (biphasic stimulation for 3 ms); drug concentrations: 0.07, 0.21, 0.7, and 2.1 µM; extracellular ion concentrations: [Na^+^] = 145 mM, [Ca^2+^] = 1.8 mM, [K^+^] = 4 mM, [Cl^−^] = 154.6 mM |
| Bepridil | (Nguyen, 2017): data collected in ventricular cardiomyocytes at 36°C and 1 Hz pacing (biphasic stimulation for 3 ms); drug concentrations: 0.032, 0.096, 0.32, and 0.96 µM; extracellular ion concentrations: [Na^+^] = 145 mM, [Ca^2+^] = 1.8 mM, [K^+^] = 4 mM, [Cl^−^] = 154.6 mM |
| Chlorpromazine | (Nguyen, 2017): data collected in ventricular cardiomyocytes at 36°C and 1 Hz pacing (biphasic stimulation for 3 ms); drug concentrations: 0.0345, 0.1035, 0.345, and 1.035 µM; extracellular ion concentrations: [Na^+^] = 145 mM, [Ca^2+^] = 1.8 mM, [K^+^] = 4 mM, [Cl^−^] = 154.6 mM |
| Cisapride | (Nguyen, 2017): data collected in ventricular cardiomyocytes at 36°C and 1 Hz pacing (biphasic stimulation for 3 ms); drug concentrations: 0.00258, 0.0258, 0.0774, 0.258 µM; extracellular ion concentrations: [Na^+^] = 145 mM, [Ca^2+^] = 1.8 mM, [K^+^] = 4 mM, [Cl^−^] = 154.6 mM |
| Clarithromycin | (Nguyen, 2017): data collected in ventricular cardiomyocytes at 36°C and 1 Hz pacing (biphasic stimulation for 3 ms); drug concentrations: 1.2, 12, 36, 120 µM; extracellular ion concentrations: [Na^+^] = 145 mM, [Ca^2+^] = 1.8 mM, [K^+^] = 4 mM, [Cl^−^] = 154.6 mM |
| Clozapine | (Nguyen, 2017): data collected in ventricular cardiomyocytes at 36°C and 1 Hz pacing (biphasic stimulation for 3 ms); drug concentrations: 0.071, 0.213, 0.71, 2.13 µM; extracellular ion concentrations: [Na^+^] = 145 mM, [Ca^2+^] = 1.8 mM, [K^+^] = 4 mM, [Cl^−^] = 154.6 mM |
| Diltiazem | (Nguyen, 2017): data collected in ventricular cardiomyocytes at 36°C and 1 Hz pacing (biphasic stimulation for 3 ms); drug concentrations: 0.128, 0.384, 1.28, and 3.84 µM; extracellular ion concentrations: [Na^+^] = 145 mM, [Ca^2+^] = 1.8 mM, [K^+^] = 4 mM, [Cl^−^] = 154.6 mM |
| Dofetilide | (Nguyen, 2017): data collected in ventricular cardiomyocytes at 36°C and 1 Hz pacing (biphasic stimulation for 3 ms); drug concentrations: 0.002, 0.02, 0.06, and 0.22 µM; extracellular ion concentrations: [Na^+^] = 145 mM, [Ca^2+^] = 1.8 mM, [K^+^] = 4 mM, [Cl^−^] = 154.6 mM |
| Domperidone | (Nguyen, 2017): data collected in ventricular cardiomyocytes at 36°C and 1 Hz pacing (biphasic stimulation for 3 ms); drug concentrations: 0.02, 0.2, 0.6, and 2 µM; extracellular ion concentrations: [Na^+^] = 145 mM, [Ca^2+^] = 1.8 mM, [K^+^] = 4 mM, [Cl^−^] = 154.6 mM |
| Droperidol | (Nguyen, 2017): data collected in ventricular cardiomyocytes at 36°C and 1 Hz pacing (biphasic stimulation for 3 ms); drug concentrations: 0.016, 0.048, 0.16, 0.48 µM; extracellular ion concentrations: [Na^+^] = 145 mM, [Ca^2+^] = 1.8 mM, [K^+^] = 4 mM, [Cl^−^] = 154.6 mM |
| Ibutilide | (Nguyen, 2017): data collected in ventricular cardiomyocytes at 36°C and 1 Hz pacing (biphasic stimulation for 3 ms); drug concentrations: 0.1, 0.3, 1, 3 µM; extracellular ion concentrations: [Na^+^] = 145 mM, [Ca^2+^] = 1.8 mM, [K^+^] = 4 mM, [Cl^−^] = 154.6 mM |
| Loratadine | (Nguyen, 2017): data collected in ventricular cardiomyocytes at 36°C and 1 Hz pacing (biphasic stimulation for 3 ms); drug concentrations: 0.00045, 0.00135, 0.0045, 0.0135; µM extracellular ion concentrations: [Na^+^] = 145 mM, [Ca^2+^] = 1.8 mM, [K^+^] = 4 mM, [Cl^−^] = 154.6 mM |
| Mexiletine | (Nguyen, 2017): data collected in ventricular cardiomyocytes at 36°C and 1 Hz pacing (biphasic stimulation for 3 ms); drug concentrations: 0.25, 2.5, 25, 75 µM; extracellular ion concentrations: [Na^+^] = 145 mM, [Ca^2+^] = 1.8 mM, [K^+^] = 4 mM, [Cl^−^] = 154.6 mM |
| Nifedipine | (Nguyen, 2017): data collected in ventricular cardiomyocytes at 36°C and 1 Hz pacing (biphasic stimulation for 3 ms); drug concentrations: 0.0077, 0.0231, 0.077, 0.231 µM; extracellular ion concentrations: [Na^+^] = 145 mM, [Ca^2+^] = 1.8 mM, [K^+^] = 4 mM, [Cl^−^] = 154.6 mM |
| Nitrendipine | (Nguyen, 2017): data collected in ventricular cardiomyocytes at 36°C and 1 Hz pacing (biphasic stimulation for 3 ms); drug concentrations: 0.00302, 0.00906, 0.0302, 0.0906; µM extracellular ion concentrations: [Na^+^] = 145 mM, [Ca^2+^] = 1.8 mM, [K^+^] = 4 mM, [Cl^−^] = 154.6 mM |
| Ondansetron | (Nguyen, 2017): data collected in ventricular cardiomyocytes at 36°C and 1 Hz pacing (biphasic stimulation for 3 ms); drug concentrations: 0.372, 1.116, 3.72, 11.16 µM; extracellular ion concentrations: [Na^+^] = 145 mM, [Ca^2+^] = 1.8 mM, [K^+^] = 4 mM, [Cl^−^] = 154.6 mM |
| Quinidine | (Nguyen, 2017): data collected in ventricular cardiomyocytes at 36°C and 1 Hz pacing (biphasic stimulation for 3 ms); drug concentrations: 0.3, 3, 3, 100 µM; extracellular ion concentrations: [Na^+^] = 145 mM, [Ca^2+^] = 1.8 mM, [K^+^] = 4 mM, [Cl^−^] = 154.6 mM |
| Ranolazine | (Nguyen, 2017): data collected in ventricular cardiomyocytes at 36°C and 1 Hz pacing (biphasic stimulation for 3 ms); drug concentrations: 2, 20, 60, 200 µM; extracellular ion concentrations: [Na^+^] = 145 mM, [Ca^2+^] = 1.8 mM, [K^+^] = 4 mM, [Cl^−^] = 154.6 mM |
| Sotalol | (Nguyen, 2017): data collected in ventricular cardiomyocytes at 36°C and 1 Hz pacing (biphasic stimulation for 3 ms); drug concentrations: 1.5, 15, 150, 450 µM; extracellular ion concentrations: [Na^+^] = 145 mM, [Ca^2+^] = 1.8 mM, [K^+^] = 4 mM, [Cl^−^] = 154.6 mM |
| Tamoxifen | (Nguyen, 2017): data collected in ventricular cardiomyocytes at 36°C and 1 Hz pacing (biphasic stimulation for 3 ms); drug concentrations: 0.0221, 0.0663, 0.221, 0.663 µM; extracellular ion concentrations: [Na^+^] = 145 mM, [Ca^2+^] = 1.8 mM, [K^+^] = 4 mM, [Cl^−^] = 154.6 mM |
| Vandetanib | (Nguyen, 2017): data collected in ventricular cardiomyocytes at 36°C and 1 Hz pacing (biphasic stimulation for 3 ms); drug concentrations: 0.3, 0.9, 3.0, 9.0 µM; extracellular ion concentrations: [Na^+^] = 145 mM, [Ca^2+^] = 1.8 mM, [K^+^] = 4 mM, [Cl^−^] = 154.6 mM |
| Verapamil | (Nguyen, 2017): data collected in ventricular cardiomyocytes at 36°C and 1 Hz pacing (biphasic stimulation for 3 ms); drug concentrations: 0.01, 0.1, 1.0, 10.0 µM; extracellular ion concentrations: [Na^+^] = 145 mM, [Ca^2+^] = 1.8 mM, [K^+^] = 4 mM, [Cl^−^] = 154.6 mM |
|  |  |

**Table S7.** Sources and details of the experimental biomarker data used for the evaluation of the computational drug effect translation. Sarcomere length biomarker drug effect data were collected in (**a**) rat and (**b**) human cardiomyocytes.

(**a**)

|  |  |
| --- | --- |
| **Drug** | **Source and details** |
|  |  |
|  |  |
| Thapsigargin | Data set #3: data collected in left ventricular cardiomyocytes at 35-37°C and 1 Hz pacing (monophasic stimulation for 5 ms); extracellular ion concentrations: [Na^+^] = 140 mM, [Ca^2+^] = 1.5 mM, [K^+^] = 4 mM; drug concentrations: 0.1, 1, 10 and 30 µM |
|  |  |

(**b**)

|  |  |
| --- | --- |
| **Drug** | **Source and details** |
|  |  |
|  |  |
| Thapsigargin | (Abi-Gerges et al., 2020): data collected in ventricular cardiomyocytes at 36°C and 1 Hz pacing (biphasic stimulation for 3 ms); drug concentrations: 0.1, 1, 10 and 30 µM; extracellular ion concentrations: [Na^+^] = 145 mM, [Ca^2+^] = 1.8 mM, [K^+^] = 4 mM, [Cl^−^] = 154.6 mM |
|  |  |

**References**

Asp, M.L., Martindale, J.J., & Metzger, J.M. (2013). Direct, differential effects of tamoxifen, 4-hydroxytamoxifen, and raloxifene on cardiac myocyte contractility and calcium handling. PLoS One, 8(10), e78768. https://doi.org/10.1371/journal.pone.0078768

Bell, D., & McDermott, B.J. (1995). Inhibition by verapamil and diltiazem of agonist-stimulated contractile responses in mammalian ventricular cardiomyocytes. Journal of Molecular and Cellular Cardiology, 27(9), 1977-1987. https://doi.org/10.1016/0022-2828(95)90019-5

Bokenes, J., Sjaastad, I., & Sejersted, O.M. (1985). Artifactual contractions triggered by field stimulation of cardiomyocytes. Journal of Applied Physiology, 98(5), 1712-1719. https://doi.org/10.1152/japplphysiol.00630.2004

Britton, O.J., Bueno-Orovio, A., Virág, L., Varró, A., & Rodriguez, B. (2017). The electrogenic Na+/K+ pump is a key determinant of repolarization abnormality susceptibility in human ventricular cardiomyocytes: a population-based simulation study. Frontiers in Physiology, 8, 278. https://doi.org/10.3389/fphys.2017.00278

Coppini, R., Ferrantini, C., Yao, L., Fan, P., Del Lungo, M., Stillitano, F., Sartiani, L., Tosi, B., Suffredini, S., Tesi, C., Yacoub, M., Olivotto, I., Belardinelli, L., Poggesi, C., Cerbai, E., & Mugelli, A. (2013). Late sodium current inhibition reverses electromechanical dysfunction in human hypertrophic cardiomyopathy. Circulation, 127(5), 575-584. https://doi.org/10.1161/CIRCULATIONAHA.112.134932

Høydal, M. A., Kirkeby-Garstad, I., Karevold, A., Wiseth, R., Haaverstad, R., Wahba, A., Stølen, T.L., Contu, R., Condorelli, G., Ellingsen, O., Smith, G.L., Kemi, O.J., & Wisløff, U. (2018). Human cardiomyocyte calcium handling and transverse tubules in mid-stage of post-myocardial-infarction heart failure. ESC Heart Failure, 5(3), 332-342. https://doi.org/10.1002/ehf2.12271

O’Hara, T., Virág, L., Varró, A., & Rudy, Y. (2011). Simulation of the undiseased human cardiac ventricular action potential: model formulation and experimental validation. PLoS Computational Biology, 7(5), e1002061. https://doi.org/10.1371/journal.pcbi.1002061

Williams, S., Pourrier, M., McAfee, D., Lin, S., & Fedida, D. (2014). Ranolazine improves diastolic function in spontaneously hypertensive rats. American Journal of Physiology. Heart and Circulatory Physiology, 306(6), H867-H881. https://doi.org/10.1152/ajpheart.00704.2013
