# Supplementary material for: Computational modelling for improved translation of cardiac inotropic and lusitropic drug effects from rats to humans
Source: J Pharmacol Toxicol Methods. Author manuscript; Available in PMC 2026 Jan 3. (PMC12759087; doi:10.1016/j.vascn.2025.107747)
Supplement: Supplementary material 2 [file NIHMS2122390-supplement-Supplementary_material_2.docx]

**Supplementary Figures**

**
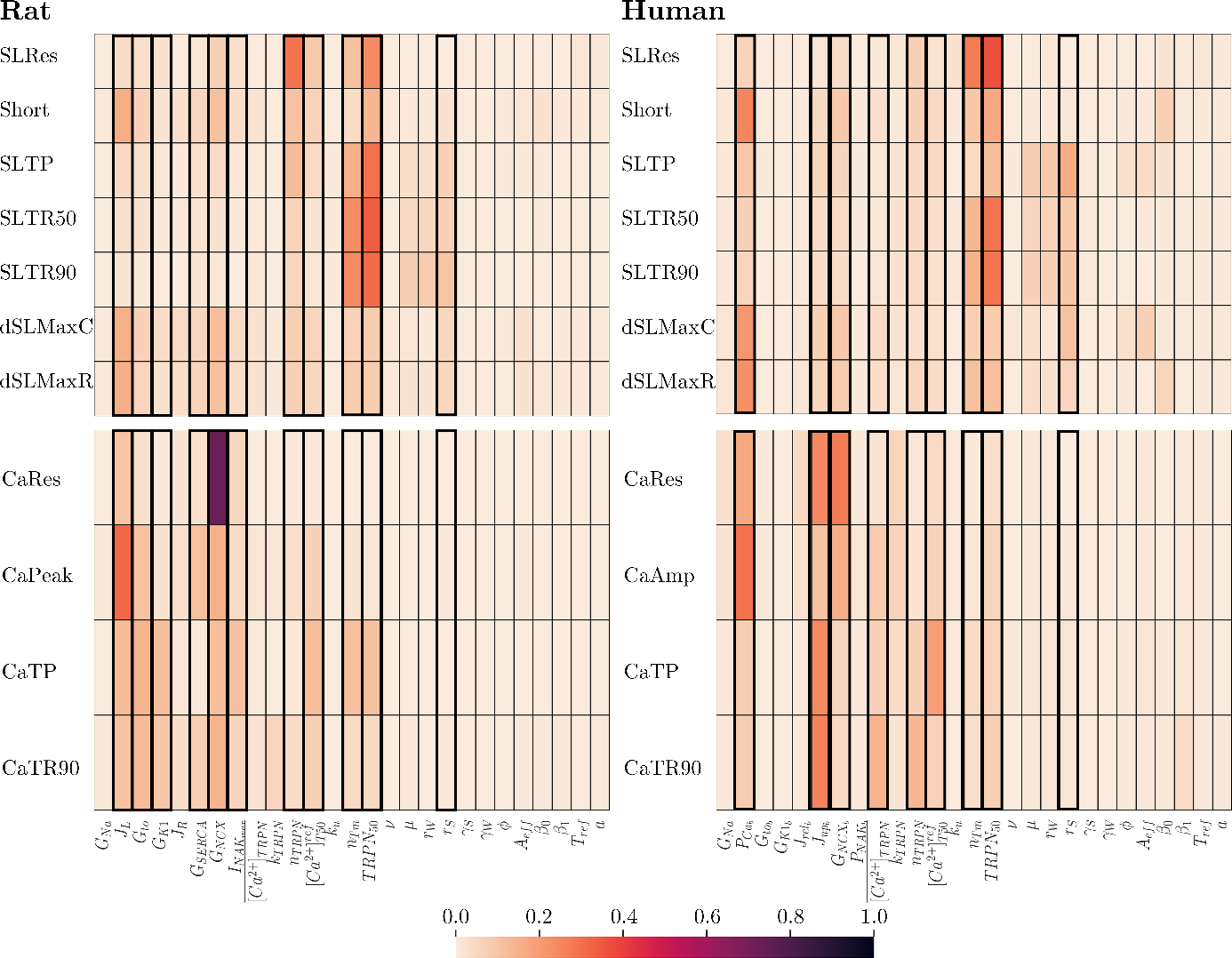
Fig. S1.** Global sensitivity analyses **to inform** the parameter selection for the rat and human cardiomyocyte model recalibration. Normalised total-effect sensitivity indices ($S_{T}^{n}$) with respect to sarcomere length and intracellular [Ca^2+^] biomarkers are shown. Bold frames indicate parameters selected for recalibration ($S_{T}^{n}$ ≥ 0.1 with respect to at least one biomarker). The proportion of excluded transients was 1.6% and 6.4%, in the analyses on the rat and human cardiomyocyte model, respectively.

**
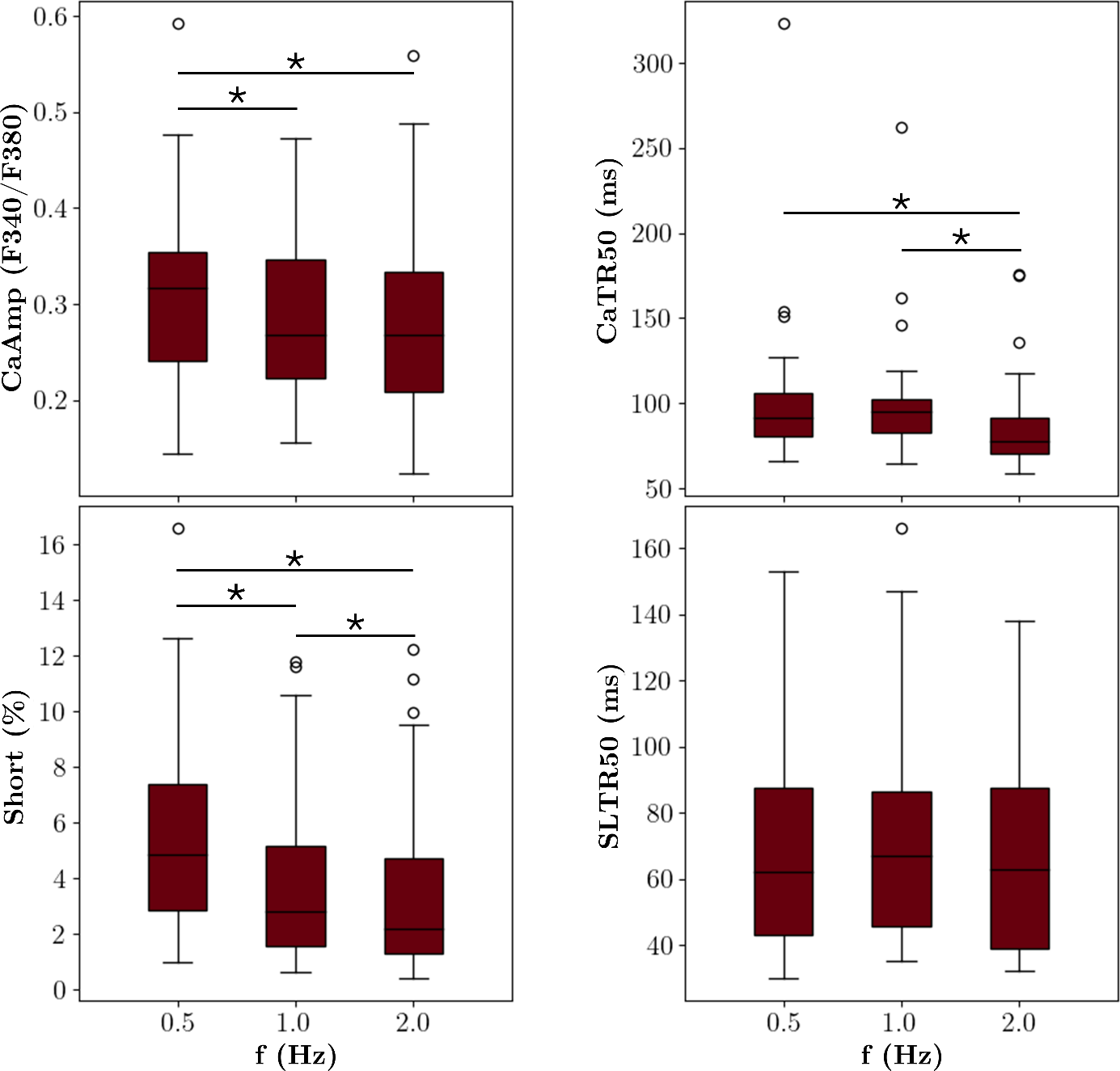
**

**Fig. S2.** Experimental frequency relationship of sarcomere length (N = 31) and intracellular [Ca^2+^] (N = 28) biomarkers. Bars with black stars on top indicate significant differences (p < 0.05). Normality was assessed using the Shapiro-Wilk test, and since most data sets were not normally distributed, the Wilcoxon rank-sum test with Holm-Bonferroni correction was applied to p-values. Statistical analyses were performed using a custom Python script.

**
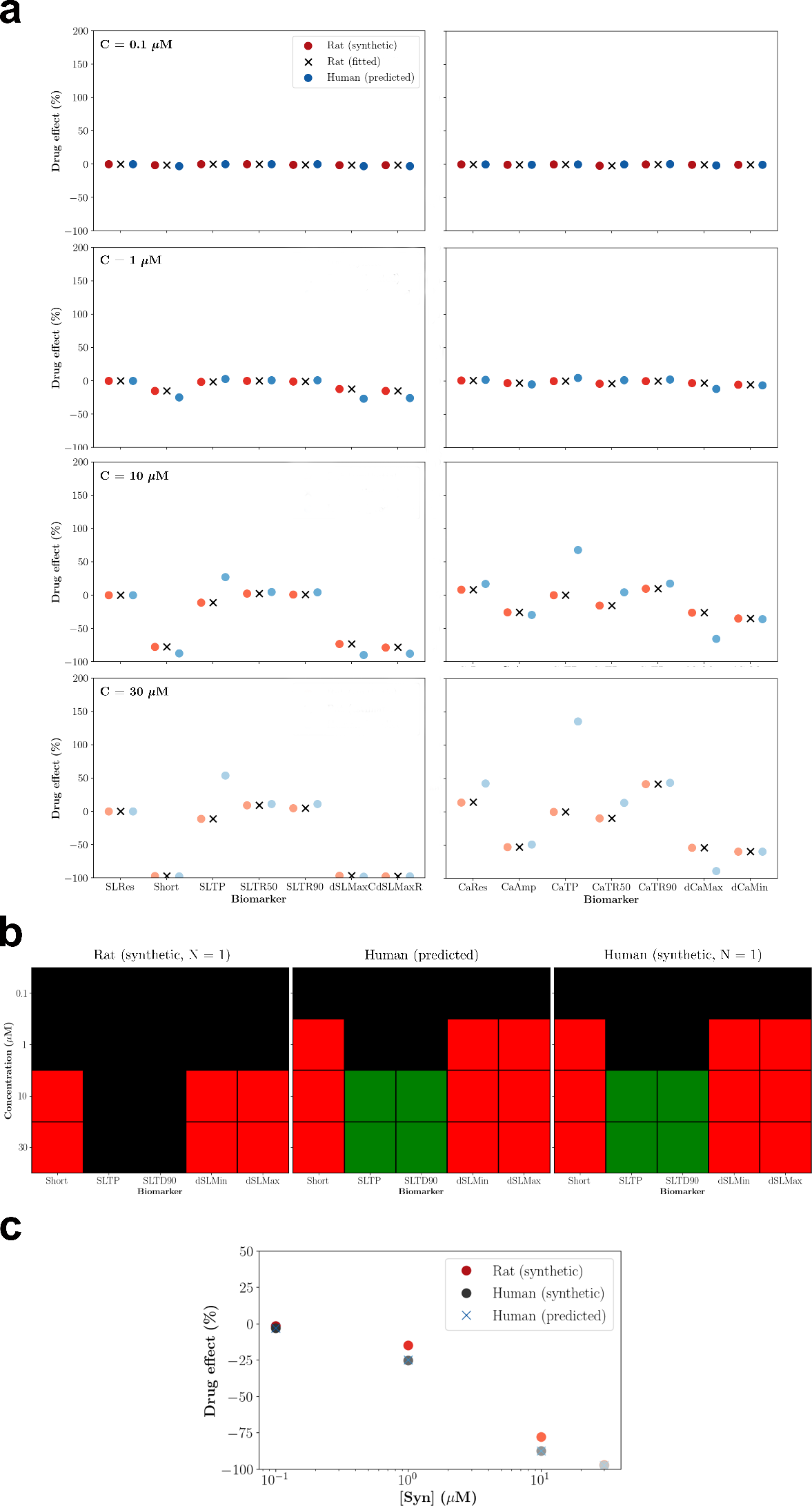
**

**Fig. S3.** Evaluation of computational drug effect translation using supplementary synthetic data generated by a virtual SERCA2 blocker (pIC50 = 4.704) administered at the same four concentrations as thapsigargin. (**a**) Synthetic rat drug effects, corresponding fitting results, and predicted human drug effects. (**b**) Qualitative comparison of experimental rat drug effects, synthetic human drug effects, and experimental human drug effects. Green, red, and black colours indicate positive (≥ 10%), negative (≤ −25%), and absent (> −25% & < 10%) drug effects, in line with the classification used in Ngyuen et al. (2017) and Abi-Gerges et al. (2020). (**c**) Quantitative comparison of synthetic rat drug effects, predicted human drug effects, and synthetic human drug effects on the percentage sarcomere shortening.
